# The pro-inflammatory signalling regulator Stat4 promotes vasculogenesis of great vessels derived from endothelial precursors

**DOI:** 10.1038/ncomms14640

**Published:** 2017-03-03

**Authors:** Zhao-Zheng Meng, Wei Liu, Yu Xia, Hui-Min Yin, Chi-Yuan Zhang, Dan Su, Li-Feng Yan, Ai-Hua Gu, Yong Zhou

**Affiliations:** 1 Key Laboratory of Stem Cell Biology, Institute of Health Sciences, Shanghai Institutes for Biological Sciences, Chinese Academy of Sciences & Shanghai Jiao Tong University School of Medicine, Shanghai 200031, China; 2 State Key Laboratory of Reproductive Medicine, Institute of Toxicology, School of Public Health, Nanjing Medical University, Nanjing 211166, China

## Abstract

Vasculogenic defects of great vessels (GVs) are a major cause of congenital cardiovascular diseases. However, genetic regulators of endothelial precursors in GV vasculogenesis remain largely unknown. Here we show that Stat4, a transcription factor known for its regulatory role of pro-inflammatory signalling, promotes GV vasculogenesis in zebrafish. We find *stat4* transcripts highly enriched in *nkx2.5*^*+*^ endothelial precursors in the pharynx and demonstrate that genetic ablation of *stat4* causes stenosis of pharyngeal arch arteries (PAAs) by suppressing PAAs 3–6 angioblast development. We further show that *stat4* is a downstream target of *nkx2.5* and that it autonomously promotes proliferation of endothelial precursors of the mesoderm. Mechanistically, *stat4* regulates the emerging PAA angioblasts by inhibiting the expression of *hdac3* and counteracting the effect of *stat1a*. Altogether, our study establishes a role for Stat4 in zebrafish great vessel development, and suggests that Stat4 may serve as a therapeutic target for GV defects.

## Abstract (web summary)

Stat4 is a transcription factor known to regulate pro-inflammatory signalling. Here, Meng *et al*. show that Stat4 is not only regulating inflammation but it is also crucial for great vessels development and endothelial precursor proliferation in zebrafish, by inhibiting the expression of *hdac3* and counteracting the effect of Stat1a.

Great vessel defects (GVDs) are one of the major causes of congenital cardiovascular diseases, which lead to the death of millions of infants and children annually^1^,^2^. GVDs include carotid artery anomalies, aberrant subclavian arteries, interrupted aortic arch, coarctation of the aorta, patent *ductus arteriosus*, proximal pulmonary artery hypoplasia and others^3^,^4^. Cellular and genetic mechanisms of great vessel malformations remain largely unknown, partly due to the intricate structure and transformation of GVs in fetal growth^5^,^6^. Investigation of the vasculogenesis of great vessels may help clarify GVD etiologies^7^. During vasculogenesis, angioblasts, which originate from endothelial precursors of the mesoderm, coalesce and assemble into the primary structure of great vessels such as the aortic arch arteries (also known as pharyngeal arch arteries, PAAs)^8^,^9^. The PAAs connect the blood flow from the heart to the dorsal aorta in the embryo, and they provide an indispensable contribution to the carotid arteries and great vessels of the heart^10^,^11^.

Recent studies have found that the endothelium of great vessels is derived from mesodermal precursors expressing heart field markers, such as Nkx2.5. It has been reported that subpopulations of *nkx2.5*^+^ cells in the anterior lateral plate mesoderm (ALPM) condense to form four pairs of clusters that give rise to PAAs 3–6, but the function of Nkx2.5 is not required until the activation of the vasculogenesis programme of great vessels^12^. This finding suggests that spatiotemporally specific mediators regulate the angioblast fate of *nkx2.5*^+^ endothelial precursors to form great vessels. We previously found that repression of the second heart field (SHF) regulator Ltbp3 led to great vessel anomalies^13^. It is reported that the deletion of the SHF regulator FGF8 in the cardiogenic mesoderm also causes PAA phenotypes in mice^14^. Tcf21, together with Nkx2.5, marks a dispensable group of PAA progenitors for great vessel formation in zebrafish^10^. Although *Tbx* haploinsufficiency causes aortic arch defects in mice, it is involved in endoderm–mesenchyme interactions but not angioblast formation in the mesoderm^15^,^16^. Despite the roles of these cardiogenic regulators in the development of great vessels, the specific regulators of *nkx2.5*^+^ endothelial precursors of great vessels have not been revealed.

Using the transparent zebrafish embryo model, we are able to visualize the process of great vessel vasculogenesis, which is difficult to study in mice and humans due to gestation in the uterus^13^,^17^. This vasculogenesis programme in zebrafish lasts from 28 h post-fertilization (hpf) to 48 hpf, which is comparable to similar processes that occur in the fourth week of human embryo development^11^,^12^. Using this model, we conduct a transcriptional microarray analysis of *nkx2.5*^+^ cells at 30 hpf and identify signal transducer and activator of transcription factor 4 (*stat4*), which is highly expressed, as a potential regulator of *nkx2.5*^+^ endothelial precursors. As a pro-inflammatory signalling mediator, Stat4 has long been shown to direct the development of fully functional T helper 1 cells and is canonically activated by interleukin (IL)-12 receptor-associated JAK kinases^18^,^19^.

In this study, we generate a *stat4* mutant line using the CRISPR/Cas system, and describe a pivotal role of Stat4 in regulating the proliferation of endothelial precursors during great vessel vasculogenesis. Furthermore, we uncover that *stat4* is downstream of *nkx2.5* and promotes angioblast formation by suppressing *hdac3* and *stat1a*. Our findings elucidate a surprising and important function of Stat4 in the establishment of great vessels.

## Results

### Conservation of Stat4 and its expression in the pharynx

**Figure 1:**
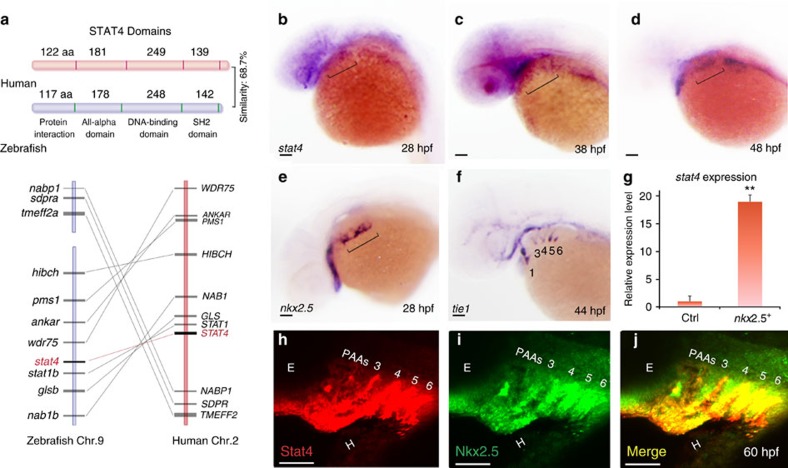
Conservation of Stat4 and its expression in *nkx2.5*^*+*^ PAA endothelial progenitors. (**a**) Schematic diagram illustrates the STAT4 protein functional domains of Human (red) and Zebrafish (blue), and synteny analysis of Stat4 loci on the human (red) and zebrafish (blue) chromosomes. The width of the lines and their distances represent the relative sizes of the genes and loci distances, respectively. (**b**–**d**) Brackets indicate that *stat4* expression resides in the pharynx at 28 hpf (**b**), 38 hpf and 48 hpf (**d**) by *in situ* hybridization. (**e**) *nkx2.5*^*+*^ cells locate in the pharynx at 28 hpf (**f**) *tie1*^*+*^ cells at 44 hpf following the appearance of *nkx2.5* pharyngeal clusters. (**g**) The expression level of *stat4* in *nkx2.5*^*+*^ cells compared with somatic cells at 30 hpf from microarray data. Error bars indicate s.d., unpaired two-tailed Student's *t*-test, **P=0.002 (triplicates for each group) (**h**,**i**) The immunohistochemistry staining of Stat4 (red) and Nkx2.5 (green) of wild-type embryos using anti-Stat4 and anti-Nkx2.5 antibodies at 60 hpf. (**j**) Co-localization of Stat4 and Nkx2.5 protein analysis by the merged image (yellow). Scale bars, 50 μm. *n*≥20 embryos per group for *in situ*, *n*=12 embryos per group for immunohistochemistry; hpf, hours post-fertilization.

The orthologues of all mammalian STAT genes in zebrafish have been identified with the high primary sequence conservation^20^. Zebrafish *stat4* encodes a 731-aa protein, which contains four functional domains and shares 68.7% sequence similarity with human STAT4. *Stat4* is located on Chromosome 9 in zebrafish, and no paralogs have been identified. The genes flanking zebrafish *stat4* are syntenic with the *STAT4* locus in humans. As evident from the domain structure and amino-acid sequence alignment, zebrafish *stat4* is closely related to the corresponding mammalian homologues (Fig. 1a and Supplementary Fig. 1).

The expression level of *stat4* was found to be 18-fold higher in *nkx2.5*^*+*^ cells by transcriptional microarray analysis of the Tg(*nkx2.5*:Zsyellow) line at 30 hpf than in somatic cells (Fig. 1g). Pathway enrichment analysis showed that Jak-Stat signalling was a major upregulated pathway in *nkx2.5*^*+*^ cells (Supplementary Fig. 2A). The activated genes of the Jak-Stat pathway were listed in the heatmap and scatter plot, among which *stat4* was the most highly activated (Supplementary Fig. 2B,C). *In situ* hybridization showed that expression of *stat4* was enriched in the ALPM at 28 h post fertilization (hpf) and 48 hpf (Fig. 1b–d and Supplementary Fig. 3A–G). *Stat4* transcripts were also distributed in the pharyngeal arch area at 60 hpf (Supplementary Fig. 3H,I). As shown in Supplementary Fig. 3E–I, *stat4* is not expressed in the heart at 24 hpf or 48 hpf, but we could observe the weak expression of *stat4* in the heart at 60 hpf.

Similar to *stat4*, *nkx2.5* transcripts were co-localized in the pharyngeal clusters of the ALPM at 28 hpf, which subsequently differentiates into aortic arch angioblasts in the pharynx, as visualized by the angioblast marker *tie1* (Fig. 1e,f). Later, *nkx2.5*^+^ ZsYellow populations from the mesoderm formed the primitive great vessels, the third to sixth pairs of PAAs (PAAs 3–6) visualized by the ZsYellow^+^/mCherry^+^ population of Tg(*nkx2.5*:ZsYellow);Tg(*flk1*:mCherry) embryos (Supplementary Fig. 4A–C). The Stat4 protein co-localized with Nkx2.5 proteins in PAAs 3–6 at 60 hpf, as revealed by double immunofluorescent staining (Fig. 1h–j).

### Loss of *stat4* causes stenosis of the primitive great vessels

A splice morpholino, targeting the splice acceptor site for intron 3 of *stat4*, was employed to suppress normally spliced *stat4* transcripts in zebrafish embryos, generating *stat4* morphants. Reverse transcription PCR showed that the transcript was efficiently knocked down. Whereas control embryos expressed normal *stat4* transcripts, the morphants expressed improperly spliced *stat4* messenger RNAs (mRNAs) without exons 3 and 4 (Supplementary Fig. 5A). The *stat4* morphants did not show obvious developmental abnormalities other than the stenosis of PAAs 3–6.

**Figure 2:**
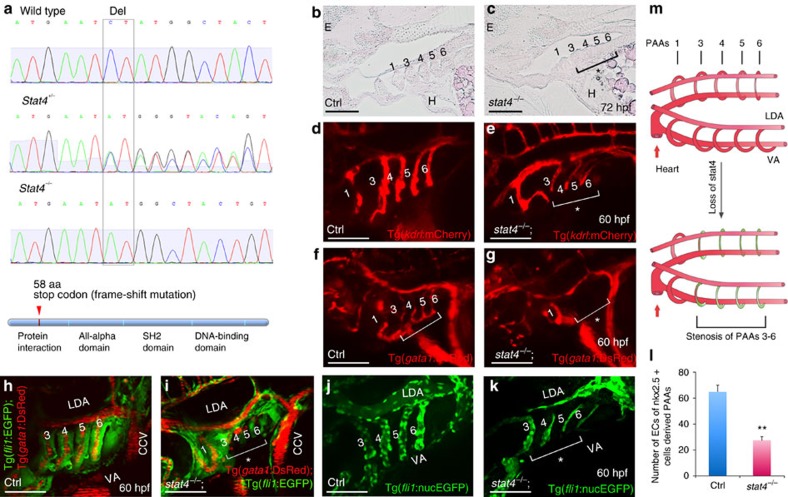
Loss of *stat4* leads to stenosis of PAAs 3–6. (**a**) Genomic sequences of wild-type zebrafish, *stat4* heterozygous mutants and homozygous mutants. The *stat4* mutant has a two-base pair deletion, which causes a frame-shift mutation and a premature stop codon to form a truncated protein with 58 amino acids. (**b**,**c**) Pathological sections (10 μm per section) of wild-type and mutant embryos with Haematoxylin-Eosin staining at 72 hpf (*n*=12). (**d**,**e**) Confocal images of PAAs in the control Tg(*kdrl*:mCherry) embryos and the *stat4*^−/−^;Tg(*kdrl*:mCherry) line (*n*=9). (**f**,**g**) Images of PAAs 3–6 vessels trafficking blood flow in the control and *stat4*^−/−^;*Tg(gata1:DsRed)* embryos (*n*=9). (**h**,**i**) Images of PAAs trafficking blood flow in the control and the *stat4*^−/−^;Tg(*fli1*:EGFP);Tg(*gata1*:DsRed) line (*n*=8). (**j**–**l**) Control and the *stat4*^−/−^;Tg(*fli1:*nucEGFP) embryos show PAA endothelial cells (green) with cell number counts at 60 hpf (*n*=9). Error bars indicate s.d., unpaired two-tailed Student's *t*-test, **P=0.003. (**m**) The model of PAAs 3–6 phenotype caused by *stat4* deficiency is illustrated in the cartoon. Scale bars, 50 μm. LDA, lateral dorsal aorta; VA, ventral aorta; E, eye; H, heart. The brackets with asterisks highlight the malformed individual PAAs.

The CRISPR/Cas system was employed to generate *stat4* zebrafish mutants by targeting exon 3 (Supplementary Fig. 5B). After CRISPR/Cas RNA injection, we detect the F1 generation heterozygotes and found robust induction of targeted insertion/deletion mutations by assessing the frequency of altered alleles (Supplementary Table 1). The selected homozygous *stat4* mutant of the F3 generation shown in Fig. 2 had a two-base pair deletion that caused a frame-shift mutation and a premature stop codon in exon 3, leading to a truncated protein with 58 amino acids (Fig. 2a). The heterozygous and homozygous *stat4* mutants were born at normal Mendelian ratios and showed atrophic pharyngeal regions and a slightly small head at 72 hpf compared with the wild-type siblings. The mutants had fewer and shorter arch arteries than their wild-type siblings, which have full-length arch arteries and plump necks. There were no apparent defects in the other tissues or the development of homozygotes (Supplementary Fig. 6A–C). The *stat4* mutant and morphant embryos had the same phenotype, but the deficiency of mutants was stronger than that of morphants.

The defect of PAAs 3–6 can be seen in pathological sections of the mutants. The arch arteries in mutants lack lumen endothelium, but the surrounding smooth muscle of the vasculature did not have any evident abnormality compared with wild-type controls. The deficiency of PAAs 3–6 in the mutants led to atrophy of the pharynx, and the jaw defect can also be observed in tissue sections (Fig. 2b,c). The stenosis of PAAs 3–6 caused by *stat4* deficiency can be clearly visualized by the *stat4*^−/−^;Tg(*kdrl*:mCherry) lines (Fig. 2d,e). Furthermore, the shunted blood flow labelled by *gata1*^+^ was undetectable in PAAs 3–6 vessels in the *stat4*^−/−^;Tg(*gata1*:DsRed) line, while the major vessels such as the lateral dorsal aorta and common cardinal vein retained robust blood flow (Fig. 2f,g). The *stat4*^−/−^;Tg(*fli1*:EGFP);Tg(*gata1*:DsRed) line indicated that the narrow and short PAAs 3–6 in the mutants were unable to support blood flow (Fig. 2h,i and Supplementary Fig. 7A). The lack of blood flow was due to stenosis of the arch artery lumens, visualized in the *stat4*^−/−^;Tg(*fli1*:nucEGFP) line. The cell count numbers revealed an ∼58% decrease in differentiated endothelial cells of PAAs 3–6 in *stat4* mutants compared with the control (Fig. 2j-l). As shown in Supplementary Fig. 7B,C, the yellow cells remained in PAAs 3–6 of *stat4* morphants in the Tg (*nkx2.5*:zsyellow);Tg(*kdrl*:mCherry) line, indicating that the remained structure of PAAs 3–6 was derived from *nkx2.5*^+^ cells. However, an insufficient number of *nkx2.5*^+^ cells led to the deficient growth of the arch arteries in the morphants compared with the controls.

Taken together, suppression of *stat4* caused agrowth deficiency of PAAs 3–6, while the first pair of aortic arch arteries that is established before the initiation of circulation were not impaired in either the control or *stat4* mutant/morphant embryos. The model of stenosis of the primitive great vessels (PAAs 3–6) caused by *stat4* deficiency is shown in Fig. 2m.

### Lack of Stat4 prevents emergence of PAA angioblasts

**Figure 3:**
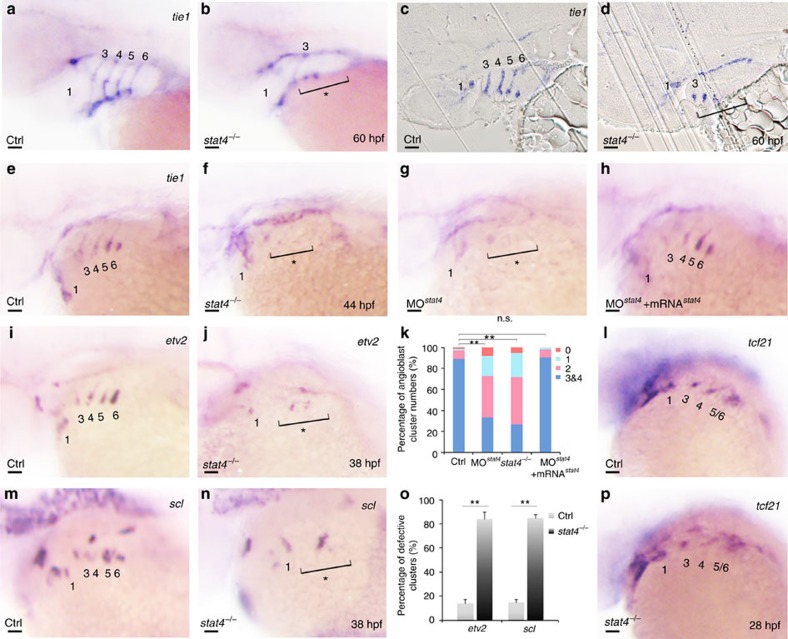
Lack of *stat4* represses emergence of PAA angioblasts. (**a**,**b**) *tie1* transcripts expressing PAAs are visualized in the control and *stat4* mutants at 60 hpf. (**c**,**d**) Resin sections (10 μm per section) of wild-type and *stat4* mutant embryos with *tie1 in situ* hybridization at 60 hpf. (**e**–**h**) Analysis of *tie1* expression in pharyngeal angioblastic cords is conducted in the control embryos (**e**), *stat4* mutants (**f**), *stat4* morphant embryos (**g**) and embryos with *stat4* mRNA and *stat4* morpholino injection (**h**) at 44 hpf by *in situ* hybridization. (**k**) Proportional quantification of embryos with defective PAA angioblast cords in *tie1* staining assays, Kruskal–Wallis test with the Dunn's multiple comparison test, **P<0.01, n.s.: *P*>0.05. (**i**,**j**,**m**,**n**) The *etv2* and *scl* transcripts are evaluated by *in situ* hybridization in the control and *stat4* mutants at 38 hpf. (**o**) Percentage of embryos with defective PAA angioblastic cords in *etv2* and *scl* staining assays, error bars indicate the s.d., Kruskal–Wallis test, **P=0.0058, *n*=30 per each group. (**l**,**p**) *tcf21*^+^ PAAs are visualized in the control and *stat4* mutants at 28 hpf. Scale bars, 50 μm. **a**–**d**, *n*=23 embryos in each group; **e**–**h**, *n*=32 embryos in each group; **l**,**p**, *n*=26 embryos per group.

In *stat4* mutants, the lack of *tie1* transcripts in arch arteries 3–6 of the *stat4* mutants suggested a decline in the vasculogenesis programme of the *nkx2.5*^+^ cells derived from PAAs 3–6 compared with the control group at 60 hpf (Fig. 3a,b). The resin sections showed that the *tie1* probe labelled the four cords of PAAs angioblasts in the control, while only minimal *tie1*^*+*^ angioblasts were observed in PAAs 3–6 of *stat4* mutants (Fig. 3c,d). The vasculogenesis defect in *stat4* mutants was observed as early as emergence of the four pairs of angioblastic cords (ACs) (as indicated by the asterisk and bracket). Loss of *stat4* inhibited the emergence of *tie1*-labelled ACs, the number of which was significantly decreased in mutants at 44 hpf. The *stat4* morphants phenocopied the *tie1*^+^ PAA angioblast deficiency seen in the mutants. The recruitment of *stat4* mRNA specifically restored all four pairs of ACs in the morphants (Fig. 3e–h,k). Evaluation of endothelial precursors by *etv2* and *scl* at the top of the signalling hierarchy of angioblast formation demonstrated that suppression of *stat4* inhibited the emergence of both *etv2* and *scl* clusters in *stat4* mutants (Fig. 3i,j,m–o). The myoblast marker *tcf21* was not affected in the mutants at 28 hpf (Fig. 3l,p) or in the Tg (*tcf21*:GFP) *stat4* morphants at 30 hpf (Supplementary Fig. 8S,T). Evaluation of *flk1* expression, the endothelial marker, showed the loss of ACs 3–6 at 38 hpf (Supplementary Fig. 7D–F). Angiogenesis represented by *flk1*^+^ inter-segmental vessels was not affected in the *stat4* morphants (Supplementary Fig. 7G–I). Evaluation of the vascular mesoderm by *etv2* demonstrated that other vasculatures such as the cranial^21^ or somatic vasculatures were not significantly impaired by suppression of *stat4* (Supplementary Fig. 7J,K).

Embryonic haematopoiesis in the intermediate cell mass was not evidently disturbed by *scl* probe detection in morphants (Supplementary Fig. 8A,E). The *gata1*-labelled somatic blood flow was normal in *stat4* morphants compared with the control group (Supplementary Fig. 8B,F). *Rag1*-labelled T cells of the thymus and the *foxn1*-labelled thymus epithelia are not interrupted in *stat4* morphants compared with the control embryos (Supplementary Fig. 8C,D,G,H). Neural crest-derived pharyngeal mesenchyme is unaffected in *stat4* morphants, as revealed by *hand2* and *dlx2a* expression patterns (Supplementary Fig. 8I,J,M,N). The normal *tbx1* expression pattern in morphants demonstrated that the loss of *stat4* did not alter pharyngeal ectoderm or endoderm at 28 hpf, comparable to the controls (Supplementary Fig. 8K,O). Additionally, *tbx1* expression in the pharyngeal mesoderm is not significantly impaired in the mutants at 28 hpf (Supplementary Fig. 8Q,R).

Meanwhile, the *gata4*^+^ cardiac progenitors in the ALPM remain normal in *stat4* morphants (Supplementary Fig. 8L,P) at 18-somite stage (ss). Both the *nkx2.5*^+^ cardiac progenitors and the formation of the linear heart tube are normal in *stat4* morphants (Supplementary Fig. 9A,B,D,E). No obvious differences are found in the *ltbp3*^+^ SHF of the control embryos and the morphants (Supplementary Fig. 9I,L). The heart morphology from 48 hpf to 60 hpf is not impaired in the embryos lacking *stat4* compared with the control using the Tg(*nkx2.5*:ZsYellow) line (Supplementary Fig. 9C,F).

### Stat4 promotes proliferation of *nkx2.5* ^*+*^ endothelial precursors

**Figure 4:**
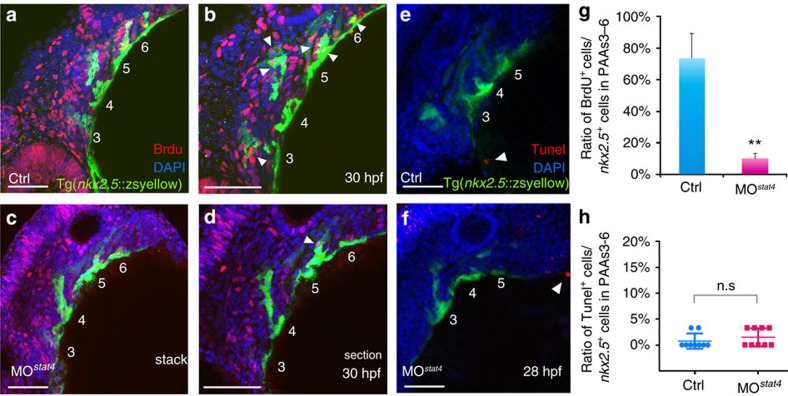
Lack of *stat4* inhibited proliferation of *nkx2.5* PAA progenitors but not apoptosis. (**a**–**d**) The control (**a**,**b**) and *stat4* morphant (**c**,**d**) Tg(*nkx2.5:*ZsYellow) embryos at 30 hpf are stained by BrdU (red), 4',6-diamidino-2-phenylindole (DAPI) (blue) as well as immunohistochemistry for ZsYellow (yellow). The single confocal sections are shown in **b**,**d**. (**e**,**f**) Projections of the pharyngeal arch region of 28 hpf control (**e**) and *stat4* morphant (**f**) Tg(*nkx2.5*:ZsYellow) embryos are assayed for TUNEL (red) and pharyngeal arch arteries for ZsYellow (yellow). (**g**) Ratios of BrdU^+^ cells/the *nkx2.5*^+^ cells in PAAs 3–6 region across three experimental replicates (*n*=6 embryos/replicate in each group); Error bars indicate the s.d. Kruskal–Wallis test, **P=0.0036. (**h**) Ratios of TUNEL^+^ cells/the *nkx2.5*^+^ cells in PAAs 3–6 region, *n*=9 per each group, n.s.: *P*>0.05. White arrowheads indicate BrdU^+^ or TUNEL^+^ cells. Scale bars, 50 μm.

Morphogenesis of the four clusters of *nkx2.5*^+^ endothelial precursors in the ALPM remained unchanged, despite the lack of *stat4*, as shown in the transverse sections of Tg(*nkx2.5:*ZsYellow) embryos at 30 hpf (Fig. 4a,c). However, the BrdU cooperation assay showed that the quantity of the co-localized BrdU^+^ (red) and *nkx2.5*^+^ (green) cells was significantly reduced in the *stat4* morphants compared with the control embryos (Fig. 4a–d,g yellow). Cellular apoptotic evaluation of *nkx2.5*^+^ clusters by TdT-mediated dUTP nick end labelling (TUNEL) revealed that the lack of *stat4* did not impair endothelial precursor survival (Fig. 4e,f,h).

**Figure 5:**
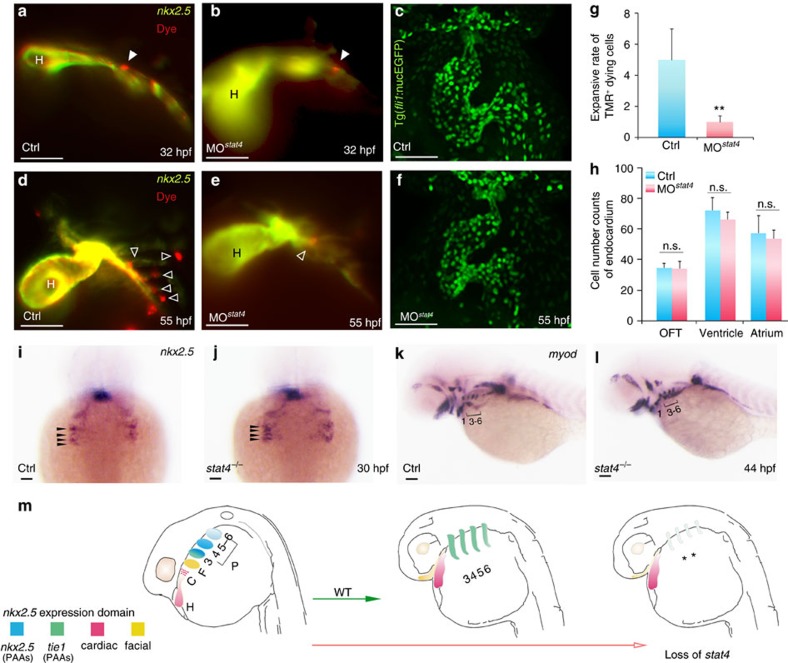
Cell tracing of endothelial precursors. (**a**,**b**,**d**,**e**) The control (**a**,**d**) and *stat4* morphant (**b**,**e**) Tg(*nkx2.5*:ZsYellow) embryos injected with CellTracker Red (white arrowheads) in the non-heart ZsYellow region at 28 hpf are imaged at 32 hpf (**a**,**b**) and again at 55 hpf (**d**,**e**). The hollow arrowheads indicate the labelled cells. (**c**,**f**) Confocal Z-stacks captured *fli1*^*+*^ endothelial cells in the heart region at 55 hpf in the control (**c**) and *stat4* morphants (**f**), respectively. (**g**) Quantification of TMR^+^ cells (*n*=12 per each group, Error bars indicate the s.d. Kruskal–Wallis test, **P=0.003). (**h**) Quantification of green endothelial cells at 55 hpf across three experimental replicates (*n*=6 embryos/replicate in each group. Analysis of variance (ANOVA) test with multiple comparison *post-hoc* test, *P=*0.9999 no significant (n.s.) difference in the outflow tract (OFT), *P=*0.6866 (n.s.) in the ventricle, *P=*0.9878 (n.s.) in the atrium between the control and *stat4* morphants. (**i**,**j**) Morphogenesis of the four pairs of *nkx2.5*^*+*^ pharyngeal clusters between the wild-type control and *stat4* mutants at 30 hpf (indicated by black arrows). (**k**,**l**) The *myod* transcripts expressing PAAs are visualized in the control and *stat4* mutants at 44 hpf. (**m**) The schematic illustration shows the fate of *nkx2.5*^+^ cells in ALPM in wild-type (WT) and loss of *stat4* embryos at 28 and 55 hpf. The first pair gives rise to the facial lineage, and 2 to 4 pairs had endothelial fate. ALPM *nkx2.5*^+^ clusters (H,C,F, PAAs 3–6). Blue: *nkx2.5* expressing cells, Green: *tie1* expressing cells, Red: cardiac lineage and Yellow: facial lineage. C: cardiac, F: facial, H: heart. Scale bars, 50 μm. *n*≥30 embryos per each group in **i**–**l**.

Cell-dye was used to label the *nkx2.5*^+^ endothelial precursors to further investigate their proliferation and migration in the absence of *stat4.* Tg(*nkx2.5:*ZsYellow) embryos were injected with CellTracker Red in the ZsYellow^+^ pharyngeal cluster region at 28 hpf. After culturing for 4 h, the single cell was labelled and imaged at 32 hpf as the starting point. At this time, the labelled cells in the control and morphant embryos did not show any obvious differences. The same embryos were imaged again at 55 hpf. As shown in Fig. 5a,b,d,e,g, the labelled cell forms multiple dye^+^ cells in the PAAs of control embryos, while the labelled cell remains in its original location without proliferation in the *stat4* morphants, indicating repressed proliferation and migration of endothelial precursors in *stat4* morphants.

We also wondered whether *stat4* regulated the fate equilibrium between the endocardium and great vessel endothelium derived from *nkx2.5*^+^ endothelial precursors. No labelled cells migrated to the heart tube in morphants, as shown in Fig. 5e. Using the Tg(*fli*: nucEGFP) line, we counted the endocardium cell numbers in the heart and outflow tract at 55 hpf and found no obvious changes in cell numbers in the morphants compared with the controls (Fig. 5c,f,h). Hence, *stat4* was not involved in the fate determination between the great vessel endothelium and the endocardium. Evaluation of *myod* indicated no defects in the PAA- and facial-muscle lineage or in the somatic skeletal muscle of mutants (Fig. 5k,l). Only the distance between PAA- muscles and the ventral aorta muscles in *stat4* mutants was shorter than that in the control embryos.

The specification of endothelial precursors in the ALPM was tested by comparing the morphogenesis of the four pairs of *nkx2.5*^*+*^ pharyngeal clusters between the controls and mutants. No evident change was found by 30 hpf between the two groups, suggesting that the specification of *nkx2.5*^*+*^ endothelial precursors was not prevented by the suppression of *stat4* (Fig. 5i,j), consistent with the results in Fig. 4a–d and Supplementary Fig. 9G,H,J,K. The schematic illustration in Fig. 5m shows the fate of *nkx2.5*^+^ cells in the ALPM, and the loss of *stat4* can inhibit the proliferation of *nkx2.5*^+^ endothelia precursor clusters 3–6 to cause insufficient assembly of the primitive great vessels.

### Stat4 acts downstream of Nkx2.5 to regulate PAA angioblasts

**Figure 6:**
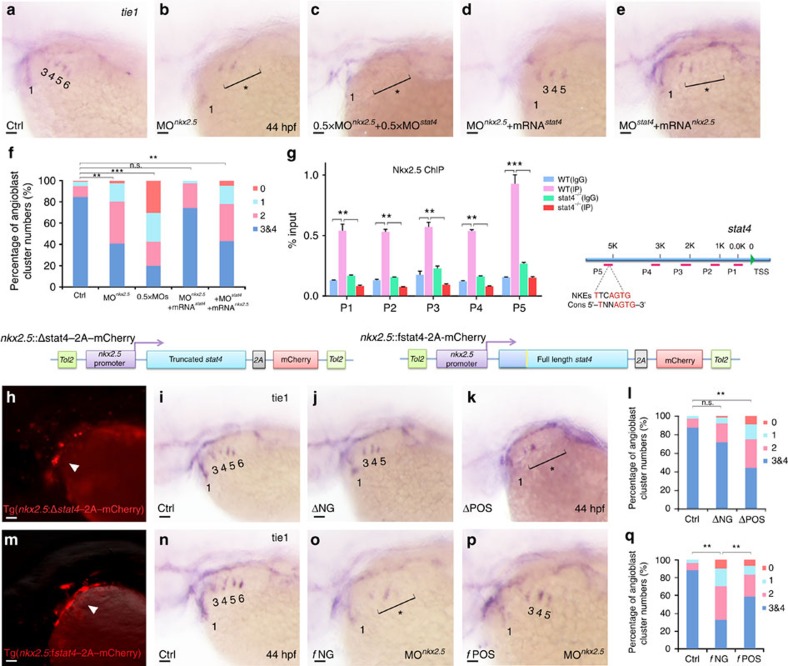
Cell-autonomous requirement of Stat4 downstream of Nkx2.5. (**a**–**e**) *In situ* hybridization analysis of *tie1* transcripts in the control (**a**), *nkx2.5* morphant embryos (**b**), embryos with half doses of *nkx2.5* and *stat4* morpholinos (**c**), *nkx2.5* morphants with *stat4* mRNA (**d**) and *stat4* morphants with *nkx2.5* mRNA (**e**). (**f**) Proportional quantification of embryos with defective angioblastic cords, Kruskal–Wallis test with the Dunn's multiple comparison test, **P<0.01, ***P<0.001, n.s.: *P*>0.05, *n*≥30 per each group. (**g**) The ChIP analysis with anti-Nkx2.5 antibody for the promoter regions of *stat4* in the wild-type and *stat4* mutants at 44 hpf across six experimental replicates (*n*=30 embryos/replicate in each group, Kruskal–Wallis test with the Dunn's multiple comparison test, **P<0.01, ***P<0.001). The binding enrichment region at the stat4 locus is indicated by primers (P1–P5). The minimal DNA-binding consensus (Cons) for Nkx2.5 contains a 5'-TNNAGTG-3' sequence motif. (**h**,**m**) Cellular autonomous analysis of *stat4* is carried out by utilizing an *nkx2.5* promoter to generate chimeric embryos with transient truncated (Δ) and full-length (*f*) *stat4* gene expression in *nkx2.5*^*+*^ cells. The schematic diagram shows *tol2*-mediated transient transgenesis of *nkx2.5* promoting mCherry expression. (**h**) Specific mCherry fluorescence expresses as pharyngeal clusters in the transgenic embryos with *nkx2.5* promoter driving variant N-terminal 51 amino-acids residues truncated zebrafish *stat4.* (**m**) Exclusive mCherry fluorescence in the pharynx is observed in the transgenic *nkx2.5* promoter driving full-length wild-type form of zebrafish *stat4* in *nkx2.5* morphants embryos. (**i**–**k**) *tie1*^*+*^ PAA angioblastic cords are appraised by *in situ* hybridization in the controls (**i**), negative mCherry fluorescence expressing embryos (ΔNG) (**j**) and positive mCherry fluorescent embryos (ΔPOS). (**l**) Proportional quantification data. Kruskal–Wallis test with the Dunn's multiple comparison test, **P<0.01, n.s.: *P*>0.05, *n*≥30 per each group. (**n**–**p**) The control (**n**), negative mCherry fluorescence expressing embryos with *nkx2.5* MO (*f*NG) (**o**) and positive mCherry fluorescence expressing embryos with *nkx2.5* MO (*f*POS) (**p**). (**q**) Proportional quantification data, Kruskal–Wallis test with the Dunn's multiple comparison test, **P<0.01, *n*≥30 per each group. Scale bars, 50 μm.

Notably, *nkx2.5* knock down reduced the number of PAAs ACs, which showed a similar phenotype as the *stat4* morphant embryos, consistent with the previous study^12^. Co-suppression of *stat4* and *nkx2.5* by half dosages of morpholinos caused a total depletion of ACs 3–6. Overexpression of *nkx2.5* transcripts by injecting *in vitro* synthesized full-length *nkx2.5* mRNA did not restore the *tie1*^*+*^ AC quantity of *stat4* morphants. In contrast, replenishment of *stat4* mRNA rescued the reduction in the number of ACs in *nkx2.5* morphant embryos, as shown by the increase in the percentage of embryos with over two pairs of ACs 3–6 to control levels (Fig. 6a–f).

The chromatin immunoprecipitation (ChIP) assay showed that Nkx2.5 can bind to the promoter region of *stat4*, and the binding can be inhibited by *nkx2.5* knock down. The Nkx2.5 DNA-binding site (NKE)^22^ was found in the binding enrichment region at the *stat4* locus using primer 5, suggesting that *stat4* was a direct target of Nkx2.5 (Fig. 6g).

Cellular autonomous analysis of Stat4 was carried out by utilizing an *nkx2.5* promoter and the *tol2* transposon to generate chimeric embryos with transient gene expression in *nkx2.5*^*+*^ cells, as shown in Fig. 6h,m. Driven by the *nkx2.5* promoter, full-length (*f*) or N-terminal truncated forms (Δ) of Stat4 were expressed with high fidelity in *nkx2.5*^*+*^ cells, as indicated by the mCherry fluorescence, within bilateral ACs at 32 hpf. The *tie1 in situ* hybridization of the positive transgenic embryos (ΔPOS) with dominant-truncated Stat4 in *nkx2.5*^*+*^ cells and the negative embryos (ΔNG) reveals that ΔPOS embryos displayed a reduced number of ACs compared with ΔNG and controls. Overexpression of full-length Stat4 in *nkx2.5*^*+*^ cells (*f*POS) can sufficiently reverse the reduced number of ACs caused by the *nkx2.5* morpholino (*f*NG) (Fig. 6h–q).

### Stat4 regulates angioblasts by suppressing Stat1a and Hdac3

**Figure 7:**
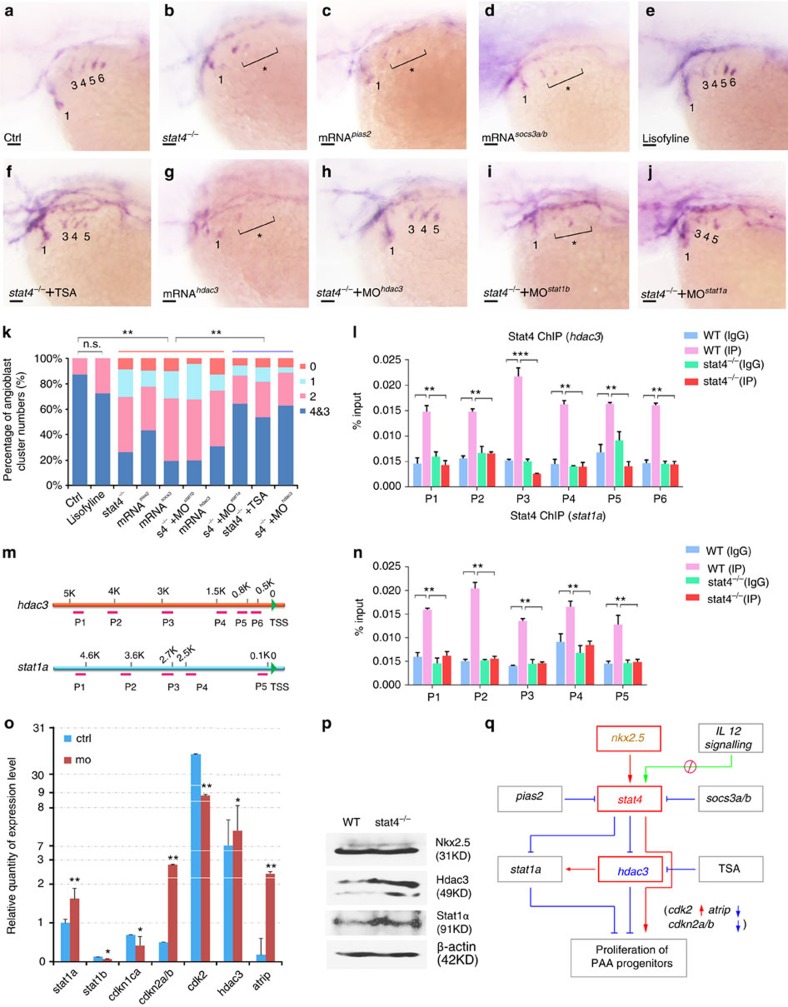
*stat4* promoted the PAA angioblast programme by suppressing *stat1a* and *hdac3.* (**a**–**k**) *In situ* hybridization analysis of *tie1* transcripts in the control (**a**), *stat4* mutants (**b**), embryos with 50 ng μl^−1^ *pias2* mRNA injection (**c**), embryos with 50 ng μl^−1^ *socs3a* and *socs3b* mRNAs injection (**d**), embryos treated with 85 μM lisofylline (**e**), *stat4* mutants with 0.2 μM TSA treatment (**f**), embryos with 50 ng μl^−1^ *hdac3* mRNA injection (**g**), *stat4* mutants with 4 ng μl^−1^ *hdac3* morpholino (**h**), *stat4* mutants with 4 ng μl^−1^ *stat1b* morpholino (**i**), *stat4* mutants with 4 ng μl^−1^ *stat1a* morpholino (**j**) at 44 hpf. Scale bars, 50 μm. (**k**) Proportional quantification of indicated *tie1*^+^ expressing cords number (*s4*^−/−^: *stat4* mutants), Kruskal–Wallis test with the Dunn's multiple comparison test, **P<0.01, n.s.: *P*>0.05, *n* ≥30 embryos per each group. (**l**–**n**) The ChIP analysis with anti-Stat4 antibody for the promoter regions of *hdac3* and *stat1a* in the wild-type control and *stat4* mutants at 44 hpf across six experimental replicates (*n*=30 embryos/replicate in each group, analysis of variance (ANOVA) test with multiple comparison *post-hoc* test, **P<0.01, ***P<0.001). (**m**) The binding enrichment regions at the *hdac3* and *stat1a* locus are indicated by primers (P1–P6). (**o**) Q-PCR results of related genes expression in the control and *stat4* morphants at 44 hpf across six experimental replicates (*n*=10 embryos/replicate in each group. Error bars indicate s.d., unpaired two-tailed Student's *t*-test, *P<0.05, **P<0.01. (**p**) Protein levels of Nkx2.5, Hdac3 and Stat1a in the wild-type control and *stat4* mutants detected by western blots at 44 hpf. β-actin is used as the internal control. (**q**) A network is drawn to illustrate the *stat4* pathway in controlling PAA proliferation. Red: activation, Blue: suppression.

To understand the downstream signalling of Stat4 in this process, a small molecule lisofylline, the canonical inhibitor of IL-12-mediated *stat4* activation^23^, was used to treat the embryos. The *tie1 in situ* hybridization of lisofylline-treated embryos showed that the number of PAA angioblast cords did not decline, indicating that IL-12 signalling was not involved in the angioblast formation. However, overexpression of the Stat4-specific inhibitor Socs3a/b^24^ did reduce the number of *tie1*-expressing cords. Similarly, overexpression of another Stat4-specific inhibitor, Pias2 (previously known as Piasx^25^), also resulted in a decrease of *tie1*-expressing cords, mimicing the phenotype of *stat4* mutants (Fig. 7a–e,k).

Many studies have confirmed the association of the Stat4-Stat1 locus in chronic inflammation diseases^26^, and Stat4 can counteract the function of Stat1 in T cells^27^. Hence, we knocked down *stat1a* and *stat1b* in *stat4* mutants and found that the knockdown of *stat1a* can partially rescue the loss of ACs but not *stat1b* (Fig. 7i,j). It has been reported that Hdac3 deacetylates Stat1, thus permitting its phosphorylation and ability to inhibit proliferation. Notably, HDAC type I and II inhibitor Trichostatin A (TSA)-treated embryos rescues the PAA angioblast cluster loss seen in mutants. It has been reported that HDACs 3 is responsive to TSA treatment in vascular endothelium^28^. We found that the injection of *hdac3* mRNA can efficiently inhibit vasculogenesis of the great vessels. Moreover, morpholino-mediated *hdac3* knock down can rescue the PAAs phenotype of the *stat4* mutant (Fig. 7f–h,k).

The ChIP assays showed that Stat4 can target the promoter regions of *hdac3* and *stat1a*, and the binding was mostly enriched in the *hdac3* promoter region identified by primer 3 (Fig. 7l–n). Furthermore, the levels of Hdac3 and Stat1 proteins were upregulated in *stat4* mutants, as shown in the western blot, while the Nkx2.5 protein level was not affected (Fig. 7p and Supplementary Fig. 10). Expression level changes of these genes were confirmed by quantitative PCR (Q-PCR) results. The cell proliferation-related gene *cdk2* was significantly downregulated, whereas the expression levels of its inhibitors *cdkn2a/b* and *atrip* were elevated in the absence of *stat4* (Fig. 7o). Based on the described results, we show a schematic representation of the signalling pathway regulated by *stat4* and involved in PAA angioblast formation (Fig. 7q).

## Discussion

The deficiency of primitive great vessels is a key predisposing factor for cardiovascular disease^29^,^30^. Unlike the relatively advanced knowledge of endothelial development, little information about the regulation of endothelial precursors of the great vessels has been available to date. Our findings demonstrate that Stat4 has a critical role in promoting vasculogenesis of the great vessels. Several lines of evidence support this conclusion: (1) *stat4* transcripts are located specifically in the pharynx and persisted throughout PAA vasculogenesis; (2) loss of *stat4* selectively causes the stenosis of PAAs 3–6 by suppressing angioblast emergence of the great vessels in mutants; (3) lack of *stat4* inhibits the proliferation but not the apoptosis, fate equilibrium or specification of *nkx2.5*^+^ endothelial precursors; (4) Stat4 expression is regulated by Nkx2.5 to autonomously promote PAA angioblast formation; and (5) Stat4 promotes PAA angioblast emergence by inhibiting epigenetic regulator *hdac3* expression and counteracting the effects of *stat1a*.

PAA endothelial progenitors derive from the caudal *nkx2.5*^+^ clusters in the ALPM^12^. We found that the proliferation of these progenitors is Stat4 dependent. The *nkx2.5*^+^ PAA endothelial progenitors could still differentiate into arch artery endothelium without Stat4. However, lack of *stat4* prevents proliferation of *nkx2.5*^+^ cells and eventually results in stenosis of the primitive GVs. A previous study showed that *tcf21*, together with *nkx2.5*, marked a group of PAA progenitor cells, but the *tcf21*^+^ *nkx2.5*^+^ progenitors were not required for PAA formation^10^. In this study, we found that genetic ablation of *stat4* did not affect the *tcf21*-expressing PAA cells. The PAA- and facial-muscle also remained normal in *stat4* mutants, indicating that the muscle cell lineage of PAAs was not affected by the loss of *stat4*. This evidence implicated that *stat4* might specifically regulate the PAA endothelial cell lineage rather than other cell lineages of PAAs.

Although Stat4 has been most extensively investigated as a critical mediator for several pro-inflammatory cytokines and chemokines such as IL-12 and type I interferon in T lymphocytes, it was unknown whether it had any role in vasculogenesis. Several studies have provided clues that the Stat4-mediated inflammation is involved in cardiovascular diseases. STAT4 has been demonstrated to be one of the strongest genetic susceptibility factors for systemic lupus erythematosus, and notably, patients with systemic lupus erythematosus with the *STAT4* risk allele have a strikingly increased risk of cardiovascular disease^31^. *Stat4* has been found to be involved in the development of cardiac allograft vasculopathy in mice^32^. Also, PIAS2, the specific inhibitor of STAT4, has a critical role in human umbilical vein endothelial cells^33^. This evidence implicates a genetic predisposition via *STAT4* to some cardiovascular diseases.

Some of the Stat4-related pro-inflammatory factors may participate in the vasculogenesis of great vessels. Recent studies have highlighted a paradigm where the endogenous mechanisms of pro-inflammatory factors function to maintain normal tissue homeostasis^34^. Pro-inflammatory cytokines and chemokines are also reported to be expressed in the hearts of infants with congenital heart disease with GVDs^35^. Low levels of *STAT4* are expressed in cultured human umbilical vein endothelial cells and are tyrosine-phosphorylated by interferon but not IL-12 (ref. 36). We show here that canonical IL-12-Stat4 signalling does not participate in Stat4-mediated PAA vasculogenesis, however, Stat4-specific inhibition-by Pias2, Socs3a and Socs3b suppresses PAA angioblasts formation. Most SOCS proteins act in a classical negative-feedback loop to inhibit cytokine signal transduction^37^. Socs3 can specifically inhibit the activation of Stat4 by binding to the Stat4 docking site in its upstream receptor through the SH2 domain. In addition, *socs3a* and *socs3b* are found to be highly expressed in *nkx2.5*^+^ cells, together with the upregulation of *stat4* in this study (Supplementary Fig. 2).

Since previous studies have confirmed the association of STAT4-STAT1 locus on chromosome 2 (Fig. 1a) in inflammatory diseases^18^,^26^, and *stat1a* was enriched in the *nkx2.5*^+^ cells in our microarray, we speculated that Stat1 may be involved in the Stat4 signalling network. Indeed, repression of *stat1a* can partially rescue GVDs caused by the absence of *stat4*. Previous studies have shown that *stat1* has anti-proliferation roles in pro-inflammatory cells and vasculatures^38^. Moreover, Stat4 acts as a key molecule in overcoming the Stat1-dependent inhibition of proliferation in T cells^27^. Hence, it is possible that Stat4 can activate the proliferation of PAAs by inhibiting Stat1a.

It is reported that HDAC can modulate the pro-inflammatory response^39^, thus it was conceivable to hypothesize that HDAC may be involved in the Stat4 signalling in GV vasculogenesis. Indeed, we show that treatment with the HDAC type I and II specific inhibitor TSA can reverse the PAA phenotype of *stat4* mutant embryos, and Hdac3 has an important role in PAA vasculogenesis downstream of Stat4. A recent study showed that TSA could enhance vascular repair by recruiting human endothelial progenitors and increasing the expression of SCL-dependent genes, which may support our findings here^40^. It is reported that the HDAC3 inhibitor has no significant effect on the heart^28^,^41^. We also confirmed that *stat4* deficiency did not have evident effects on the heart during PAAs formation. Meanwhile, Stat1 can be deacetylated by Hdac3, thus permitting its phosphorylation^42^. Based on our data and the previous reports, Hdac3 seems to be an important target of Stat4 in mediating the proliferation of the endothelial precursors of great vessels.

In summary, our study provides insights into a potential role of the pro-inflammatory regulator Stat4 in great vessel establishment and endothelial precursor proliferation. Therefore, Stat4 can possibly serve as a diagnostic marker or a therapeutic target for GVD.

## Methods

### Zebrafish strains maintenance

Zebrafish embryos were raised at 28.5 °C and morphologically staged. The wild-type lines AB/Tubingen, Tg(*nkx2.5*:ZsYellow), Tg(*gata1*:DsRed), Tg(*flk1*:mCherry), Tg(*fli1*:eGFP), Tg (*tcf21*: GFP) and Tg(*fli1*:nucGFP) lines have been previously reported^13^. Embryos or tail fins were collected for genotyping. All zebrafish in this study were treated in strict accordance with the recommendations in the Guide for the Care and Use of Laboratory Animals of the National Institutes of Health. The experimental protocols were approved by the Review Board on the Ethics of Animal Experiments of Institute of Health Sciences, Shanghai Institutes of Biological Sciences, Chinese Academy of Sciences (Shanghai, China).

### FAC sorting and microarray analysis

The embryos of Tg(*nkx2.5*:ZsYellow) at 30 hpf were dissociated by incubation in 1 × PBS supplemented with 0.125% dispase (Life Technologies, NY, USA) for 30 to 45 min at 28.5 °C. The cells were washed and resuspended in ice-cold 0.9 × PBS plus 5% FBS and were passed through a filter with a 40 μm pore size. Samples were sorted twice into lysis buffer via a triple-LASER fluorescence-activated cell sorting instrument (FACSAria, BD Biosciences, CA, USA) to ensure cell purities of >95%, maximal RNA integrity, and minimal loss of material. Two independent sets of purified RNA from *nkx2.5*^+^ cells and *nkx2.5*^−^ somatic cells were prepared and labelled according to the manufacturer's instructions (Affymetrix, Santa Clara, CA, US). Samples were then hybridized onto a Zebrafish Oligonucleotide Microarray (AffymetrixGeneChip). The Affymetrix(R) software and GeneSpring software7.3 were used to analyse the data and visualize differential expression, respectively.

### Construction and microinjection of CRISPR/Cas

The *stat4* target sites of CRISPR/Cas were determined online (http://zifit.partners.org/ZiFiT/CSquare9Nuclease.aspx). For the efficient induction of mutagenesis in *stat4*, one target site was finally selected from the candidate sites. *Stat4* guide RNA (gRNA) was targeted to exon 3 (CRISPR/Cas *stat4* E3, GGCATCAAACCATGAATCTATGG) with forward (5′-ATAGGCAT CAAACCATGAATCTAGT-3′) and reverse (5′-TAAAACTAGATTCATGGTTTGATGC-3′) primers. *Stat4* gRNA was obtained by *in vitro* transcription with the MAXISCRIPT T7 Kit (Ambion, Thermo Fisher Scientific Inc., US) and then purified using the *mir*Vana miRNA Isolation Kit (Ambion) following the manufacturer's instructions. CRISPR capped *nls-zCas-nls* RNA was synthesized using SP6 mMessage mMachine Transcription Kit (Ambion). A mixture of RNAs (500 ng μl^−1^ of *nls-zCas-nls* RNA and 90 ng μl^−1^ of gRNA) in 0.05% phenol red and 120 mM KCl was injected into the cellular portion of one-cell stage embryos, resulting in an ∼60% rate of deformity at 24 hpf.

### Generation of zebrafish *stat4* mutant lines

The surviving embryos after injection with CRISPR/Cas RNAs were raised to adulthood and screened for founders with germ-line mutations in the *stat4* locus. The T7 endonuclease I (T7E1) enzyme was used to screen for mutations (New England Biolabs, MA, USA) according to the manufacturer's instructions. After the outcross between the founders and wild-type zebrafish, the outcross between the F1 and the wild-type, and the subsequent incross between the heterozygous F2 generation, we obtained several mutant lines for further investigation.

### Microinjection of zebrafish embryos

Knockdown of genes was achieved by the injection of corresponding anti-sense morpholinos into one-cell-stage embryos. Morpholinos were purchased from Gene Tools (USA). *Nkx2.5* morphants were obtained via the injection of 1 nl of *nkx2.5* morpholino (4 ng μl^−1^) targeting the *nkx2.5* splicing site^43^. To generate *stat4* morphants, one-cell stage embryos were injected with 1 nl of anti-sense morpholino (6 ng μl^−1^) targeting the third-intron fourth exon splice acceptor sequence (MO^*stat4*^: 5′-GTATTTCACCTGGGAGAATAGAAGA-3′). To validate the effectiveness of MO^*stat4*^-mediated *stat4* splicing inhibition, we obtained first strand complementary DNAs (cDNAs) from control and MO^*stat4*^ embryos and performed PCR to amplify the sequence spanning MO^*stat4*^ target region with forward (5′-TTCAGCAACTGGATATTAAGTTCCTCGAA-3′) and reverse (5′-TGAACGCTGCTTCTTATCACA GCC-3′) primers. Then, agarose gel electrophoresis of the PCR products was used to visualize the bands.

Capped *tol2* transposase and *nkx2.5*, *stat4*, *socs3a*, *socs3b* and *pias2* mRNAs were synthesized *in vitro* from linearized plasmid templates by SP6 mMessage mMachine Transcription Kit (Ambion) (Supplementary Table 2). Rescue assays were carried out by co-injection of 50 ng μl^−1^ mRNA into one-cell-stage embryos. A 4.8 kb *nkx2.5* promoter was prepared from Tubigen genome using the forward (5′-GTAGACTGAGTATTCGTTGCGTTAT-3′) and the reverse (5′-TTCCTGACAACAGCCGA*-*3′) primers^44^. The N-terminal 51 amino-acid residues truncated *stat4* fragment were amplified from cDNA library with the forward primer (5′-CTAGCTAGCATGGCTACTGTACTCTTCAAT-3′) and the reverse primer (5′-CTAGCTAGCGGGTGAACTCATAGCGCTCTC-3′). The full-length and truncated *stat4* cDNA were driven by the *nkx2.5* promoter, and the constructs were injected into the cellular portion of the one-cell-stage embryos at 50 ng μl^−1^.

### RNA probe synthesis and whole mount *in situ* hybridization

Zebrafish wild-type cDNA was used for amplifying *stat4, tie1*, *nkx2.5*, *hand2*, *gata4*, *scl*, *etv2*, *flk1*, *tbx1*, *foxn1* and *rag1* sequences that were subcloned into pCS2+ vector (Supplementary Table 2). T3 RNA polymerase (Roche Applied Science, IN, USA) was used to generate RNA probes transcripts from linearized DNA templates.

Whole-mount *in situ* hybridizations were conducted using digoxygenin (DIG) labelled anti-sense RNA probes (Roche Applied Science, Mannheim, Germany). Embryos were collected and fixed in 4% paraformaldehyde. Following dehydration and rehydration in a decreasing methanol series, the embryos were digested with 10 μg ml^−1^ proteinase K and hybridized overnight at 70 °C. After blocking and washing, the embryos were stained with nitro-blue tetrazolium chloride/5-bromo-4-chloro-3′-indolyphosphate p-toluidine (Roche Applied Science, Mannheim, Germany).

### Compound inhibitor treatment

Inhibition of IL-12 signalling was performed by incubating wild-type Tubingen zebrafish embryos in 85 μM lisofylline egg water (Caymanchem) or an equivalent amount of dimethylsulphoxide (DMSO) as a control, starting at the 18-somite stage. Both DMSO and lisofylline-treated wild-type embryos were fixed for *tie1 in situ* hybridizations at 44 hpf and imaged. Similarly, 0.2 μM TSA was used to inhibit the function of HDACs type I and II starting at 28 hpf, and the treated embryos were fixed and subjected to *tie1 in situ* hybridizations at 44 hpf.

### Immunohistochemistry

Fixed embryos were rehydrated in 1 × PBS/0.5% Triton X-100 (PBSTx) and blocked with PBSTx/1% BSA/0.1% DMSO for 3 h. The embryos were then incubated with primary antibodies and secondary antibodies diluted in blocking solution for 2 h. Primary antibodies for GFP (IgG2a mouse monoclonal, Life technology), ZsYellow (anti-RCFP rabbit polyclonal antibody, Clontech), Nkx2.5 (Thermo Fisher, US) and Stat4 (Sigma, US) were incubated at a 1:50 dilution. Secondary antibodies (Alexa Fluor 488 goat α-mouse IgG, Alexa Fluor 546 goat-rabbit IgG (Invitrogen) were used at a 1:500 dilution.

### BrdU staining and TUNEL immunostaining

Control and MO^*stat4*^ Tg(*nkx2.5*:ZsYellow) embryos at 30 hpf were incubated in a pre-chilled 10 mM BrdU/15% DMSO solution on ice for 20 min, transferred to pre-warmed egg water and incubated for 15 min at 28.5 °C before fixation. The embryos were digested with 1 mg ml^−1^ Proteinase K for 30 min at room temperature. Mouse α-BrdU (Roche Applied Science, Mannheim, Germany) and α-mouse Alexa Fluor 546 (Invitrogen, Carlsbad, CA) primary and secondary antibodies were both used at dilutions of 1:500. Following antibody staining and washes, the embryos were incubated with 0.5 μg ml^−1^ 4′,6-diamidino-2-phenylindole in 1 × PBS for 10 min in the dark at room temperature. After three 1 × PBS washes, the embryos were mounted and imaged.

The TUNEL assay was performed with the In Situ Cell Death Detection Kit TMR red (Roche). The morpholino injected Tg(*nkx2.5*:ZsYellow) embryos and wild-type embryos at 28 hpf were fixed and stored in 100% methanol at −20 °C. After rehydration, Proteinase K digestion and acetone treatment, embryos were permeated with a permeabilization solution (0.5% Triton X–100, 0.1% sodium citrate in PBS) at room temperature for 1 h. Then, the embryos were stained by the enzyme solution and photographed.

### Cell tracker red labelling

Cell Tracker Red dye was purchased from Invitrogen and diluted to 10 μM in egg water. Tg(*nkx2.5*:ZsYellow) embryos at 28 hpf were injected with an 1 nl drop of dye in the pharyngeal ZsYellow region outside the heart tube. Following the injection, images were taken at ∼32 hpf and again at 55 hpf.

### Chromatin immunoprecipitation (ChIP)

Chromatin was prepared from zebrafish embryos at 44 hpf and fragmented by sonication with Fisher Scientific's Sonic Dismembrator (6% amplitude, pulse for 10 s on and 30 s off for a total sonication ‘on' time of 2 min 40 s) to produce fragments ranging from 100 to 500 bp. Then, five percent chromatin was removed from each sample and used as the input control. ChIP was performed using antibodies specific for Nkx2.5 (PA5-49431, Thermo Fisher, US), Stat4 (WH0006775M1, Sigma, US) and IgG control (2719s, Cell Signaling, US) at dilutions of 1:100, 1:100 and 1:1,000, respectively. The bound DNA and mock DNA were used for SYBR green Q-PCR analyses.

### Western blot analysis

Western blot was performed with the standard protocols. Protein was prepared from zebrafish embryos at 44 hpf and dissolved in 4 × protein SDS–polyacrylamide gel electrophoresis Loading Buffer (Takara). Nkx2.5, Stat1a and Hdac3 were detected with anti-Nkx2.5 (PA5-49431, Thermo Fisher, US), anti-Stat1a (SAB3500364, Sigma, US) and anti-Hdac3 (ab32369, Abcam, US) at the dilution of 1:1,000, followed by incubation with an anti-rabbit IgG-horseradish peroxidase antibody (ab97069, Abcam, US) at a dilution of 1:5,000. β-actin was used as the internal control using an anti-β-actin antibody (A2228, Sigma, US), followed by an anti-mouse IgG antibody (62-6520, Invitrogen, US) diluted 1:1,000 and 1:5,000 in the block solution, respectively. Full scans of all western blots are presented in Supplementary Fig. 10.

### Confocal fluorescent imaging

Whole mount transgenic embryos were prepared in 3% agarose in a glass-bottomed culture dish, and the images were captured using an Olympus FV10-ASW confocal microscope system (Olympus, Tokyo, JP)^45^. EGFP was excited with a 488-nm Argon laser and imaged through a 505-536-nm filter. ZsYellow protein was excited with a 514-nm Argon laser and imaged through a 530-nm long pass filter. DsRed was excited with a 543-nm HeNe laser and imaged through a 560-nm long pass filter. mCherry was excited with a 564-nm HeNe laser and imaged through a 595-nm filter. The cells were counted in Z-stack confocal images using the ImageJ software.

### Quantitative PCR analysis

Total RNA was reverse transcribed into cDNA with SuperScript III Reverse Transcriptase (Invitrogen). Quantitative real-time PCR (qPCR) was performed with SYBR Green (TOYOBO) on a 7900HT Fast Real-Time PCR System (Applied Biosystems). The relative RNA amount was calculated with the ΔΔCt method and normalized with *gapdh* (the primers are listed in Supplementary Table 2).

### Statistical analysis

All biological experiments were performed at least three times. Data were analysed using Graphpad Prism 6 software. Quantitative data were presented as the mean±s.e.m. The normality of data was examined by the Shapiro-Wilk test. The data with normal distributions were analysed by the unpaired Student's two-tailed *t*-test between two groups, while the analysis of variance test with the multiple comparison *post-hoc* test was used to compare three or more groups. The data of non-normal distributions were analysed by the Kruskal–Wallis test with adjustments for multiple comparisons^46^. Statistical significance was denoted by *P<0.05, **P<0.01, ***P<0.001. The number of ACs (1 to 4) on the same side of the pharynx was counted to evaluate the deficiency of PAAs 3–6. Dysmorphic animals due to unknown developmental delays were excluded equally from the control and experimental groups.

### Data availability

Microarray data have been deposited in ArrayExpress with the accession number E-MTAB-5406. All relevant data supporting the findings of this study are available within the paper and its supplementary information files, or from the authors on request.

## Additional information

**How to cite this article:** Meng, Z.-Z. *et al*. The pro-inflammatory signalling regulator Stat4 promotes vasculogenesis of great vessels derived from endothelial precursors. *Nat. Commun.* **8,** 14640 doi: 10.1038/ncomms14640 (2017).

**Publisher's note:** Springer Nature remains neutral with regard to jurisdictional claims in published maps and institutional affiliations.

## Supplementary Material

Supplementary Information Supplementary Figures and Supplementary Tables.

## References

[b1] Global Burden of Disease Study C. Global, regional, and national incidence, prevalence, and years lived with disability for 301 acute and chronic diseases and injuries in 188 countries, 1990–2013: a systematic analysis for the Global Burden of Disease Study 2013. Lancet 386, 743–800 (2015). 26063472 10.1016/S0140-6736(15)60692-4 PMC4561509

[b2] van der Linde D. . Birth prevalence of congenital heart disease worldwide: a systematic review and meta-analysis. J. Am. Coll. Cardiol. 58, 2241–2247 (2011). 22078432 10.1016/j.jacc.2011.08.025

[b3] Srivastava D. Genetic assembly of the heart: implications for congenital heart disease. Annu. Rev. Physiol. 63, 451–469 (2001). 11181963 10.1146/annurev.physiol.63.1.451

[b4] Berguer R. Function and Surgery of the Carotid and Vertebral Arteries 1st edn LWW (2013).

[b5] Brown M. L. . Coarctation of the aorta: lifelong surveillance is mandatory following surgical repair. J. Am. Coll. Cardiol. 62, 1020–1025 (2013). 23850909 10.1016/j.jacc.2013.06.016

[b6] Congdon E. D. Transformation of the aortic-arch system during the development of the human embryo. Contrib. Embryol. 14, 49–U47 (1922).

[b7] Graham A. Development of the pharyngeal arches. Am. J. Med. Genet. A 119A, 251–256 (2003). 12784288 10.1002/ajmg.a.10980

[b8] De Val S. & Black B. L. Transcriptional control of endothelial cell development. Dev. Cell 16, 180–195 (2009). 19217421 10.1016/j.devcel.2009.01.014 PMC2728550

[b9] Schmidt A., Brixius K. & Bloch W. Endothelial precursor cell migration during vasculogenesis. Circ. Res. 101, 125–136 (2007). 17641236 10.1161/CIRCRESAHA.107.148932

[b10] Nagelberg D. . Origin, specification, and plasticity of the great vessels of the heart. Curr. Biol.: CB 25, 2099–2110 (2015). 26255850 10.1016/j.cub.2015.06.076 PMC4546555

[b11] Moore K. L., Persaud T. V. N. & Torchia M. G. The Developing Human: Clinically Oriented Embryology 10th edn Saunders (2015).

[b12] Paffett-Lugassy N. . Heart field origin of great vessel precursors relies on nkx2.5-mediated vasculogenesis. Nat. Cell Biol. 15, 1362–U1225 (2013). 24161929 10.1038/ncb2862 PMC3864813

[b13] Zhou Y. . Latent TGF-beta binding protein 3 identifies a second heart field in zebrafish. Nature 474, 645–648 (2011). 21623370 10.1038/nature10094 PMC3319150

[b14] Watanabe Y. . Role of mesodermal FGF8 and FGF10 overlaps in the development of the arterial pole of the heart and pharyngeal arch arteries. Circ. Res. 106, 495–U413 (2010). 20035084 10.1161/CIRCRESAHA.109.201665 PMC2843098

[b15] Zhang Z. . Tbx1 expression in pharyngeal epithelia is necessary for pharyngeal arch artery development. Development 132, 5307–5315 (2005). 16284121 10.1242/dev.02086

[b16] Vokes S. A. & Krieg P. A. Endoderm is required for vascular endothelial tube formation, but not for angioblast specification. Development 129, 775–785 (2002). 11830576 10.1242/dev.129.3.775

[b17] Hiruma T., Nakajima Y. & Nakamura H. Development of pharyngeal arch arteries in early mouse embryo. J. Anat. 201, 15–29 (2002). 12171473 10.1046/j.1469-7580.2002.00071.x PMC1570898

[b18] Korman B. D., Kastner D. L., Gregersen P. K. & Remmers E. F. STAT4: genetics, mechanisms, and implications for autoimmunity. Curr. Allergy Asthma Rep. 8, 398–403 (2008). 18682104 10.1007/s11882-008-0077-8 PMC2562257

[b19] Watford W. T. . Signaling by IL-12 and IL-23 and the immunoregulatory roles of STAT4. Immunol. Rev. 202, 139–156 (2004). 15546391 10.1111/j.0105-2896.2004.00211.x

[b20] Gorissen M., de Vrieze E., Flik G. & Huising M. O. STAT genes display differential evolutionary rates that correlate with their roles in the endocrine and immune system. J. Endocrinol. 209, 175–184 (2011). 21330334 10.1530/JOE-11-0033

[b21] Proulx K., Lu A. & Sumanas S. Cranial vasculature in zebrafish forms by angioblast cluster-derived angiogenesis. Dev. Biol. 348, 34–46 (2010). 20832394 10.1016/j.ydbio.2010.08.036

[b22] Ferdous A. . Nkx2-5 transactivates the Ets-related protein 71 gene and specifies an endothelial/endocardial fate in the developing embryo. Proc. Natl Acad. Sci. USA 106, 814–819 (2009). 19129488 10.1073/pnas.0807583106 PMC2630085

[b23] Coon M. E., Diegel M., Leshinsky N. & Klaus S. J. Selective pharmacologic inhibition of murine and human IL-12-dependent Th1 differentiation and IL-12 signaling. J. Immunol. 163, 6567–6574 (1999). 10586050

[b24] Tortorella C. . Impaired interleukin-12-dependent T-cell functions during aging: role of signal transducer and activator of transcription 4 (STAT4) and suppressor of cytokine signaling 3 (SOCS3). J. Gerontol. A Biol. Sci. Med. Sci. 61, 125–135 (2006). 16510856 10.1093/gerona/61.2.125

[b25] Arora T. . PIASx is a transcriptional co-repressor of signal transducer and activator of transcription 4. J. Biol. Chem. 278, 21327–21330 (2003). 12716907 10.1074/jbc.C300119200

[b26] Remmers E. F. . STAT4 and the risk of rheumatoid arthritis and systemic lupus erythematosus. N. Engl. J. Med. 357, 977–986 (2007). 17804842 10.1056/NEJMoa073003 PMC2630215

[b27] Gil M. P. . Regulating type 1 IFN effects in CD8 T cells during viral infections: changing STAT4 and STAT1 expression for function. Blood 120, 3718–3728 (2012). 22968462 10.1182/blood-2012-05-428672 PMC3488885

[b28] Rafehi H. . Vascular histone deacetylation by pharmacological HDAC inhibition. Genome Res. 24, 1271–1284 (2014). 24732587 10.1101/gr.168781.113 PMC4120081

[b29] van der Bom T. . The changing epidemiology of congenital heart disease. Nat. Rev. Cardiol. 8, 50–60 (2011). 21045784 10.1038/nrcardio.2010.166

[b30] Khairy P. . Changing mortality in congenital heart disease. J. Am. Coll. Cardiol. 56, 1149–1157 (2010). 20863956 10.1016/j.jacc.2010.03.085

[b31] Svenungsson E. . A STAT4 risk allele is associated with ischaemic cerebrovascular events and anti-phospholipid antibodies in systemic lupus erythematosus. Ann. Rheum. Dis. 69, 834–840 (2010). 19762360 10.1136/ard.2009.115535

[b32] Koglin J., Glysing-Jensen T., Gadiraju S. & Russell M. E. Attenuated cardiac allograft vasculopathy in mice with targeted deletion of the transcription factor STAT4. Circulation 101, 1034–1039 (2000). 10704172 10.1161/01.cir.101.9.1034

[b33] Meinert C. . Identification of intracellular proteins and signaling pathways in human endothelial cells regulated by angiotensin-(1-7). J. Proteomics 130, 129–139 (2016). 26388433 10.1016/j.jprot.2015.09.020 PMC4719126

[b34] Bruneau S., Nakayama H., Woda C. B., Flynn E. A. & Briscoe D. M. DEPTOR regulates vascular endothelial cell activation and proinflammatory and angiogenic responses. Blood 122, 1833–1842 (2013). 23881914 10.1182/blood-2013-03-488486 PMC3765062

[b35] Qing M. . Intramyocardial synthesis of pro- and anti-inflammatory cytokines in infants with congenital cardiac defects. J. Am. Coll. Cardiol. 41, 2266–2274 (2003). 12821258 10.1016/s0735-1097(03)00477-7

[b36] Torpey N., Maher S. E., Bothwell A. L. & Pober J. S. Interferon alpha but not interleukin 12 activates STAT4 signaling in human vascular endothelial cells. J. Biol. Chem. 279, 26789–26796 (2004). 15087447 10.1074/jbc.M401517200

[b37] Yoshimura A., Naka T. & Kubo M. SOCS proteins, cytokine signalling and immune regulation. Nat. Rev. Immunol. 7, 454–465 (2007). 17525754 10.1038/nri2093

[b38] Fan H. B. . miR-142-3p acts as an essential modulator of neutrophil development in zebrafish. Blood 124, 1320–1330 (2014). 24990885 10.1182/blood-2013-12-545012

[b39] Shakespear M. R., Halili M. A., Irvine K. M., Fairlie D. P. & Sweet M. J. Histone deacetylases as regulators of inflammation and immunity. Trends Immunol. 32, 335–343 (2011). 21570914 10.1016/j.it.2011.04.001

[b40] Palii C. G. . Trichostatin A enhances vascular repair by injected human endothelial progenitors through increasing the expression of TAL1-dependent genes. Cell Stem Cell 14, 644–657 (2014). 24792117 10.1016/j.stem.2014.03.003

[b41] McKinsey T. A. Therapeutic potential for HDAC inhibitors in the heart. Annu. Rev. Pharmacol. Toxicol. 52, 303–319 (2012). 21942627 10.1146/annurev-pharmtox-010611-134712

[b42] Kramer O. H. . A phosphorylation-acetylation switch regulates STAT1 signaling. Genes Dev. 23, 223–235 (2009). 19171783 10.1101/gad.479209 PMC2648535

[b43] Targoff K. L., Schell T. & Yelon D. Nkx genes regulate heart tube extension and exert differential effects on ventricular and atrial cell number. Dev. Biol. 322, 314–321 (2008). 18718462 10.1016/j.ydbio.2008.07.037 PMC2752039

[b44] Grajevskaja V., Balciuniene J. & Balciunas D. Chicken beta-globin insulators fail to shield the nkx2.5 promoter from integration site effects in zebrafish. Mol. Genet. Genomics 288, 717–725 (2013). 24036575 10.1007/s00438-013-0778-0 PMC4104600

[b45] Wang L. . miR-34b regulates multiciliogenesis during organ formation in zebrafish. Development 140, 2755–2764 (2013). 23698347 10.1242/dev.092825

[b46] Mei Y. . Adult restoration of Shank3 expression rescues selective autistic-like phenotypes. Nature 530, 481–484 (2016). 26886798 10.1038/nature16971 PMC4898763

