# Supplementary material for: The pro-inflammatory signalling regulator Stat4 promotes vasculogenesis of great vessels derived from endothelial precursors
Source: Nat Commun. 2017 Mar 3;8:14640. doi: 10.1038/ncomms14640 (PMC5338034; doi:10.1038/ncomms14640)
Supplement: Supplementary Information — Supplementary Figures and Supplementary Tables. [file ncomms14640-s1.pdf]

Supplementary Information

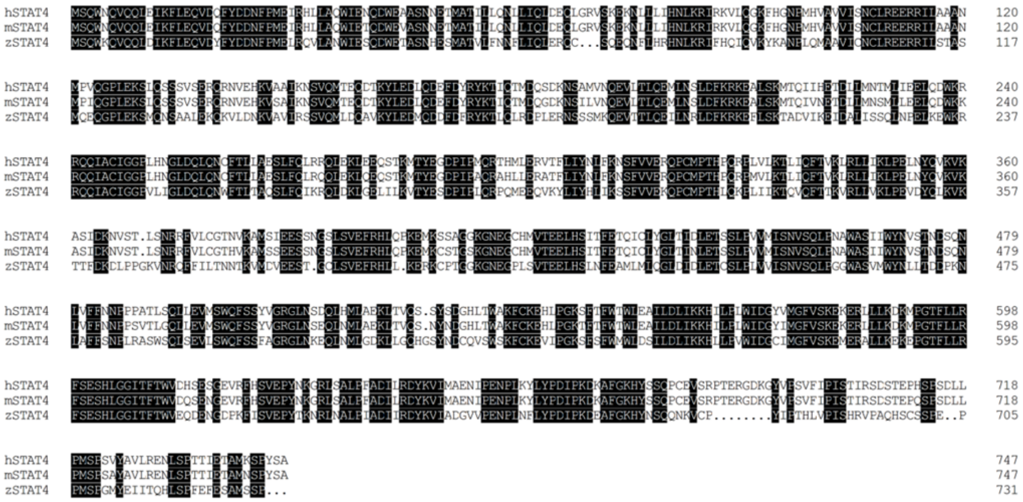

Supplementary Figure 1 Sequence alignment of human (h), mouse (m) and zebrafish (z) STAT4 proteins

Conserved residues are highlighted in the black background.

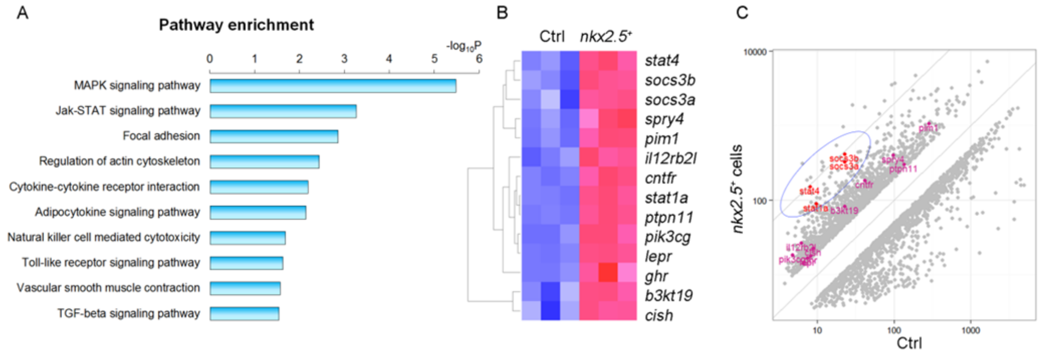

Supplementary Figure 2 The microarray screen of potential regulators of *nkx2.5*+ endothelial progenitors

(A) Pathway enrichment analysis of upregulated pathways in *nkx2.5*+ cells at 30 hpf (B) Activated genes in Jak-Stat signaling are listed in the heatmap (triplicates for each group)(C) Relative expression levels of genes in the scatter plot and the activated genes in Jak-Stat signaling labelled in red or pink.

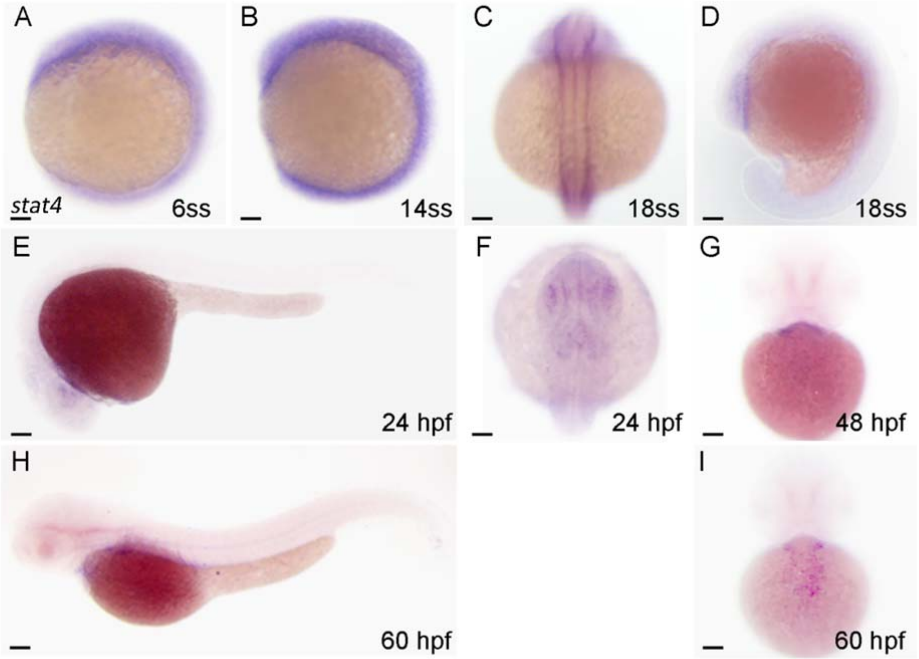

Supplementary Figure 3 Developmental expression pattern of *stat4* transcripts

(A-D) The expression of s*tat4* transcripts in zebrafish embryos at 6 ss (A), 14 ss (B) and 18 ss (C, D). (E, F) *In situ* hybridization at 24 hpf, (G) *in situ* hybridization for the expression of *stat4* at 48 hpf, and (H, I) at 60 hpf. Scale bars, 50 µm. n ≥12 each group.

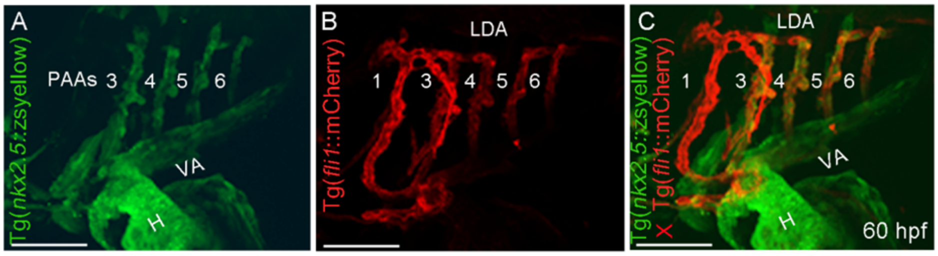

Supplementary Figure 4 PAAs 3-6 derived from *nkx2.5*$^{+}$ cells

(A) A Tg(*nkx2.5*:ZsYellow) embryo exhibits yellow fluorescence in pharyngeal arch arteries at 60 hpf. (B) Red fluorescence labels the endothelium of PAAs 1-6 in a Tg(*kdrl*:mCherry) embryo. (C) A Tg(*nkx2.5*:ZsYellow) embryo exhibiting yellow fluorescence overlapped red fluorescence in PAAs 3-6 of Tg(*kdrl*:mCherry) embryo. Scale bars, 50 µm. VA, ventral aorta; LDA, lateral dorsal aorta; H, heart. n =12.

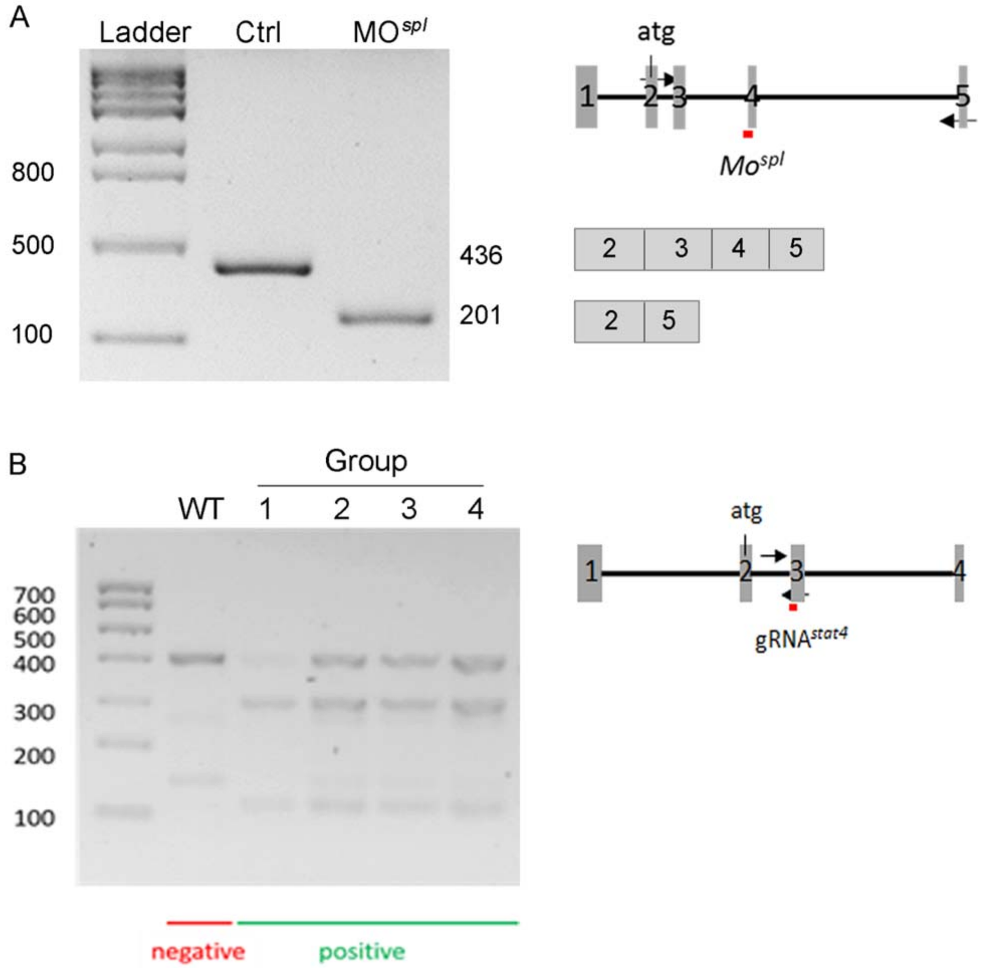

Supplementary Figure 5 Effective study of *stat4* morpholino and CRISPR/Cas mutagenesis

(A) A splice morpholino (MO^{spl}), targeting the splice acceptor site for intron 3 of *stat4*. RT-PCR shows that the control embryos express normal *stat4* transcripts, while morphants show the improperly spliced *stat4* mRNAs without exons 3 and 4. (B) A schematic diagram clarifies the target site of the *stat4* gRNA targeting exon. The T7 endonuclease I (T7EI) efficacy assay depicts the mutagenesis efficiency in *stat4* gRNA injected zebrafish embryos.

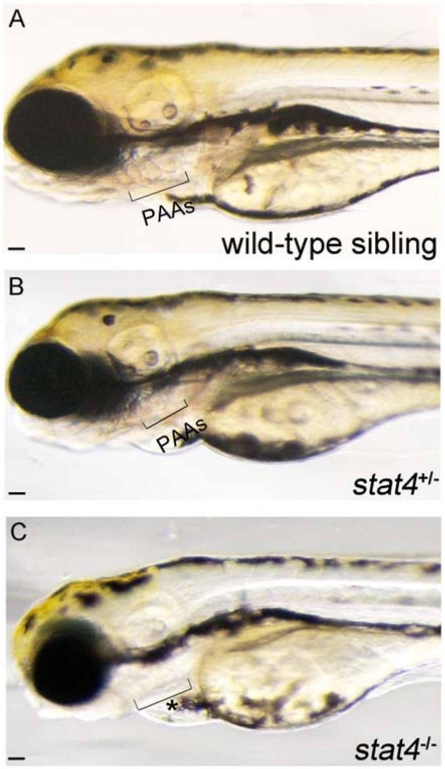

Supplementary Figure 6 The morphological phenotypes of *stat4* mutants

The wild-type siblings, the heterozygotes (*stat4*$^{+/-}$) and homozygotes (*stat4*$^{-/-}$) are imaged at 72 hpf under bright field. The pharyngeal regions and PAAs 3-6 are indicated with brackets. Scale bars, 50 µm. n ≥9 in each group.

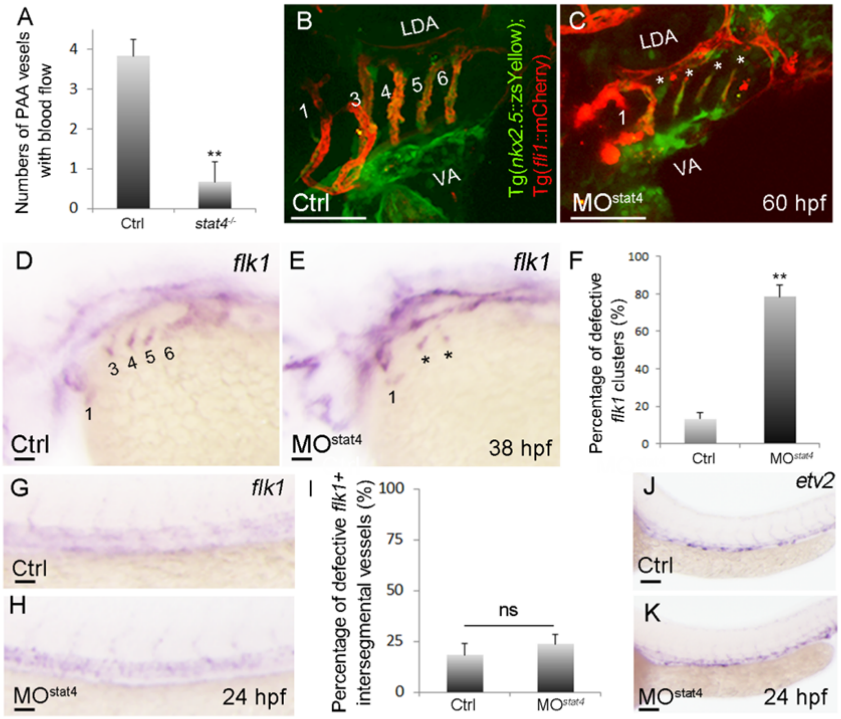

Supplementary Figure 7 Suppression of *stat4* does not affect other blood vessels and angiogenesis

(A) Quantification of PAAs numbers that support the blood flow in the control and *stat4*$^{-/-}$;Tg(*fli1*:EGFP);Tg(*gata1*:DsRed) line (n =20 per each group, Error bars indicate the standard deviation. Kruskal–Wallis test, ** *P* =0.002). (B, C) The control and *stat4* morphant embryos of the Tg(*nkx2.5:*ZsYellow);Tg(*kdrl*:mCherry) line present *nkx2.5* derivatives (yellow) and endothelial cells (red) at 60 hpf. n =20 embryos per each group (D-F) Analysis of the expression of *flk1* in pharyngeal angioblastic cords is conducted in control embryos (D) and *stat4* morphant embryos (E) at 38 hpf. (F) Percentage of embryos with defective *flk1*$^{+}$ PAA angioblastic cords in the control and *stat4* morphant embryos. n =30 per each group, Error bars indicate the standard deviation. Kruskal–Wallis test, ** *P* =0.0057 (G,H) The dorsal aorta and posterior cardinal vein as well as intersegmental vessels are assessed by *in situ* hybridization of *flk1* transcripts in the control (G) and *stat4* morphant (H) embryos at 24 hpf. (I) Quantification analysis of the defective *flk1*$^{+}$ intersegmental vessels, n =20 per each group, Error bars indicate the standard deviation. Kruskal–Wallis test, n.s.: *P* =0.37. (J, K) *In situ* hybridization of *etv2* shows the effects of *stat4* knock down on other somatic vasculatures at 24 hpf. Scale bars, 50 µm. n ≥20 each group.

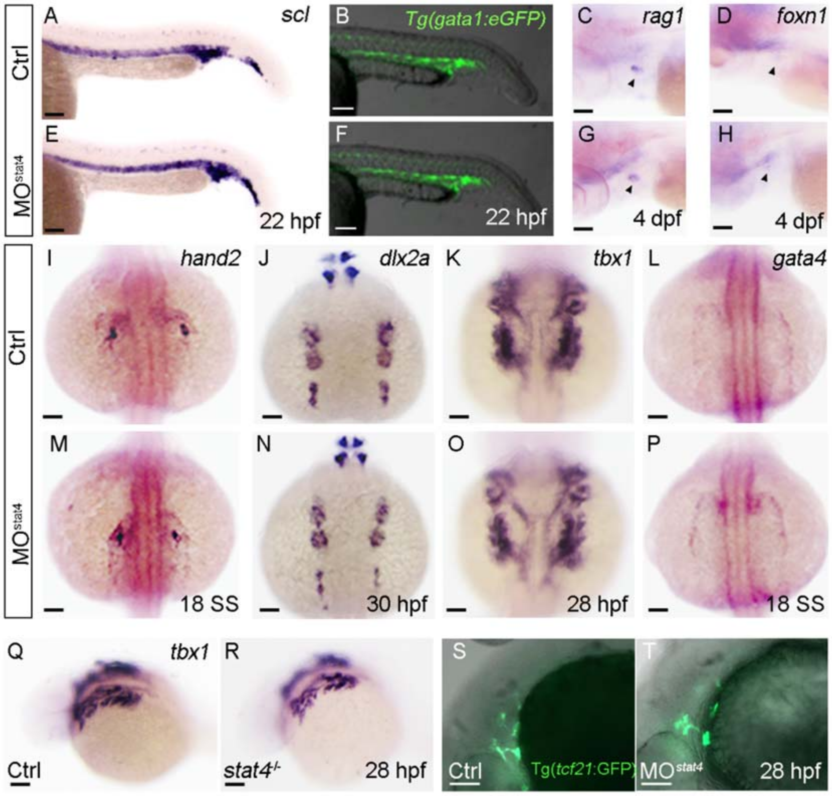

Supplementary Figure 8 Suppression of *stat4* does not affect hematopoiesis, pharyngeal mesenchyme, thymus or heart morphogenesis

(A, E) the control (A) and *stat4* morphant (E) embryos are examined by *scl in situ* hybridization for ICM at 22 hpf. (B, F) Erythroid blood flow labeled by *gata1* is imaged in the control (B) and *stat4* morphants (F) Tg(*Gata1*:EGFP) embryos at 22 hpf. (C, D, G, H) *In situ* hybridization of the T lymphocyte marker *rag1* (C, G) and thymic epithelial marker *foxn1* (D, H) in the control and *stat4* morphant embryos at 4 dpf. Arrows label T lymphocytes (C, G) and thymic epithelial cells (D, H). (I-P) The control (I, J) and *stat4* morphant embryos (M, N) are subjected to *in situ* hybridization for neural crest marker *hand2* (I, M) at 18 ss and *dlx2a* (J, N) at 30 hpf, respectively. (K, O) The control and *stat4* morphant embryos are examined by *the* expression of *tbx1* in the pharyngeal ectoderm and endoderm at 28 hpf. (L, P) Analysis of the distribution of *gata4* transcripts in the anterior lateral plate mesoderm (ALPM) at 18 ss. n ≥ 20 per each group. (Q, R) The control and *stat4* mutant embryos are examined by *in situ* hybridization of *tbx1* transcripts at 28 hpf. n =30 per each group. (S, T) Fluorescent images of PAAs in the control (Ctrl) and *stat4* morphant embryos of the Tg(*tcf21*: GFP) line at 28 hpf. Scale bars, 50 µm. n =40 per each group.

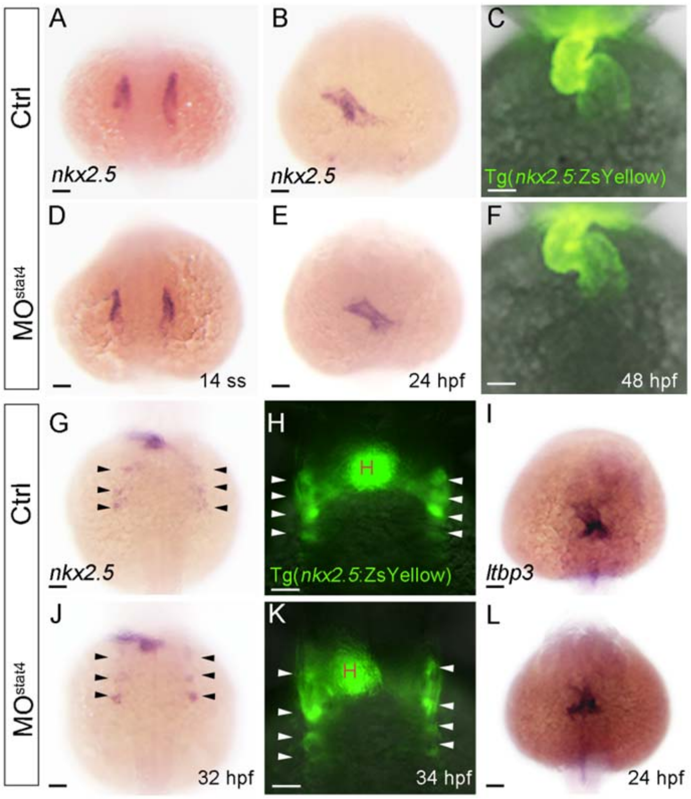

Supplementary Figure 9 Effects of *stat4* deficiency on *nkx2.5*$^{+}$ cardiac progenitors and endothelial precursors

(A, B, D, E) *In situ* hybridization detects *nkx2.5* transcripts in the control and *stat4* morphant embryos at the 14 ss (A, D) and at 24 hpf (B, E). (C, F) ZsYellow^{+} populations in the heart of the control and *stat4* morphant embryos of the Tg(*nkx2.5*:ZsYellow) line are imaged at 48 hpf. (G, J) The *nkx2.5*$^{+}$ mesodermal endothelial precursors in the control and *stat4* morphant embryos are analyzed by *in situ* hybridization at 32 hpf. (H, K) The ZsYellow^{+} population in the pharynx of the control and *stat4* morphant embryos of the Tg(*nkx2.5*:ZsYellow) line is imaged at 34 hpf. Arrowheads indicate the pharyngeal clusters. (I, L) *In situ* hybridization detects *ltbp3*$^{+}$ transcripts, which label the second heart field in the control and *stat4* morphant embryos at 24 hpf. Scale bars, 50 µm. n ≥12 in each group.

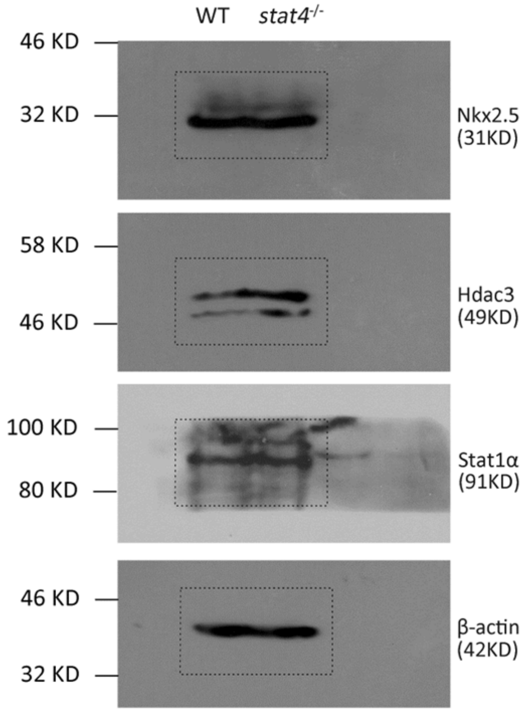

Supplementary Figure 10 Original scans of immuno-blots in the main Figure

Dotted boxes indicate the area that is shown in the main Figure 7P.

Supplementary Table 1

Alleles	Sequence	Deletion/ Insertion
stat4^{wt}	GGCTCAGGGAAACGGCATCAAACCATGAAT CTATGGCTACTGTACTCTTCAATAACTTCCTC	wt
stat4^{m1}	GGCTCAGGGAAACGGCATCAAACCATGAAT – –ATGGCTACTGTACTCTTCAATAACTTCCTC	Δ2
stat4^{m2}	GGCTCAGGGAAACGGCATCAAACCATGAAT TCCAATGGCTACTGTACTCTTCAATAACTTCCTC	Δ2+4
stat4^{m3}	GGCTCAGGGAAACGGCATCAAACCATGA– – – –ATGGCTACTGTACTCTTCAATAACTTCCTC	Δ4
stat4^{m4}	GGCTCAGGGAAACGGCATCAAACT– – – – – – – –ATGGCTACTGTACTCTTCAATAACTTCCTC	Δ8 +1
stat4^{m5}	GGCTCAGGGAAACGGCATCAAACCATGAATAACTATGGCTACTGTACTCTTCAATAACTTCCTC	+11

– Deletion, Insertion, PAM

Supplementary Table 2

Primer name	Sequence (5'- 3')	Description
spl mo F	TTCAGCAACTGGATATTAAGTTCCTCGAA	*stat4* MO evaluation
spl mo R	TGAACGCTGCTTCTTATCACAGCC	*stat4* MO evaluation
stat4 F	CCGGAATTCCCAAGTTCTGCAAGGAGGTCAT	in situ probe
stat4 R	CGGGGTACCCCTCAGCCTTTACAGACAATGG	in situ probe
hand2 F	CGGGGTACCTGCTTACTAACCGTGCCACTCT	in situ probe
hand2 R	GCTCTAGAACCATTCGCACTACGGAGGAG	in situ probe
tie1 F	CCGGAATTCGACCTTATGAGCGACCGCCATT	in situ probe
tie1 R	CGGGGTACCTTGCATCCACAGCCAAGCCTT	in situ probe
flk1 F	CACTCAATTTCAGCCCATTACT	in situ probe
flk1 R	TTCTAAGGGCCCACAAACTTTG	in situ probe
gata4 F	CCGGAATTCGGCGTCTGGACACTCTGGAT	in situ probe
gata4 R	GGGGTACCTCACTCTTGGTGCTGGAGGATC	in situ probe
nkx2.5 F	CCGGAATTCGCAAGAGGCAGCGTCAGGAT	in situ probe
nkx2.5 R	CGGGGTACCAAGTCGGAGCCGTGTTCGT	in situ probe
tbx1 F	CGGAATTCTCCAATTGCTCCTCGTCGTC	in situ probe
tbx1 R	GGGGTACCCAATTCCAGTCCGTGATAATGC	in situ probe
dlx2a F	GCTCTAGAGCTCCTGCTCCTCACCAACT	in situ probe
dlx2a R	GGGGTACCGGACACATACAAGACGGACACC	in situ probe
etv2 F	GCTCTAGAGTTCCTGCTGGTTTCGACTTC	in situ probe
etv2 R	GGGGTACCGGTTTTCTAAAGGCACCTAGCTTG	in situ probe
scl F	GATGCAACTCTAGTGCGGGACG	in situ probe
scl R	AGGAACGAGACTGCACAAATGA	in situ probe
foxn1 F	CCGGAATTCCCAAGTTCTGCAAGGAGGTCAT	in situ probe
foxn1 R	CGGGGTACCCCTCAGCCTTTACAGACAATGG	in situ probe
rag1 F	TACAGCTACAACTCTCAGCAA	in situ probe
rag1 R	AATTCCCTGTGGGACCAGTAAG	in situ probe
stat4 F	CCGGAATTCCCAAGTTCTGCAAGGAGGTCAT	mRNA

stat4 R	CGGGGTACCCCTCAGCCTTTACAGACAATGG	mRNA
nkx2.5 F	CGGAATTCATGGCAATGTTCTCTAGCCAA	mRNA
nkx2.5 R	GCTCTAGATCACCAAGCTCTGATGCCATG	mRNA
Δ stat4 F	CTAGCTAGCATGGCTACTGTACTCTTCAAT	mRNA
Δ stat4 R	CTAGCTAGCGGGTGAACTCATAGCGCTCTC	mRNA
pias2 F	ATGGCGGATATTGAGGAGTTACG	mRNA
pias2 R	TCAGTCCAGGGAGATAATGTCTG	mRNA
hdac3 F	ATGACCAATCGAACTGCGTACT	mRNA
hdac3 R	TCAGATCTCCACATCACTTTCT	mRNA
socs3a F	ATGATAACCCACAGCAAGTTGG	mRNA
socs3a R	TTAAATAGGGGCGTCATACTCCTG	mRNA
socs3b F	ATGGTAACGCATAGTAGGCTTG	mRNA
socs3b R	CTAGATGGGAGCATCGTACTCC	mRNA
ctrl MO	CCTCTTACCTCAGTTACAATTTATA	morpholino
stat4 MO	GTATTTCACCTGGGAGAATAGAAGA	morpholino
nkx2.5 MO	TGTCAAGGCTCACCTTTTTTCTCTT	morpholino
stat1a MO	GCTGAAGCTCCAACCACTGAGTCAT	morpholino
stat1b MO	GCTGGTTCCAGAGCGTCATCTTTCA	morpholino
hdac3 MO	AGAAGTACGCAGTTCGATTGGTCAT	morpholino
stat1a F	TTCGCGAGGAAAGAAAGATT	Real-time PCR
stat1a R	GGGTTCGGTGTTGGACTCT	Real-time PCR
stat1b F	TGGTCGCTACTATACCAGTGAATC	Real-time PCR
stat1b R	TGCATCTGAGTTGATGGGTTT	Real-time PCR
cdk2 F	AAAGAGCTCAATCACCCTAACA	Real-time PCR
cdk2 R	AGACGTCGAGTCCATAAACC	Real-time PCR
cdkn1ca F	TATTCGGACCCGTAGACCATG	Real-time PCR
cdkn1ca R	CAGGCAAAGGCGAGTTGGTC	Real-time PCR
cdkn2a/b F	GGATGAACTGACCACAGCAG	Real-time PCR
cdkn2a/b R	AGGCGTTCTTCTAAATTTGT	Real-time PCR

hdac3 F	AGATACACAGGTGCCTCATT	Real-time PCR
hdac3 R	ACAAAATCCAGATGCCTCAAACT	Real-time PCR
atrip F	ACGTTCATGACGAAGGAAAACT	Real-time PCR
atrip R	TCGCTGCTCTTCTGGGAGTA	Real-time PCR
gapdh F	TTGAGAAACCTGCCAAGTATGA	Real-time PCR
gapdh R	CCCATTGAAGTCAGTGGACA	Real-time PCR
stat4* P1 *F	TCAGTTCTGAACAACTTCATTGTG	ChIP
stat4* P1 *R	CATGCACCACACACTTACACC	ChIP
stat4* P2 *F	TTAGATCTCTCCTTCATTAGCGTAGA	ChIP
stat4* P2 *R	CAGGTACTGCCCTAAAACTAATGG	ChIP
stat4* P3 *F	TTCATGTGTATAACTCTCAGTGCATTA	ChIP
stat4* P3 *R	CGCTATCGATGAATATGGAAAA	ChIP
stat4* P4 *F	GGTGAAGCAGGAGCATTGAG	ChIP
stat4* P4 *R	TTACCTGTTGCCCTTTTAAGAGAC	ChIP
stat4* P5 *F	GTAGCATGATCGCAGTGAGG	ChIP
stat4* P5 *R	CACTGAAATGCCAACTGACC	ChIP
hdac3* P1 *F	GAATTGCCTCATGTGCTTTTT	ChIP
hdac3* P1 *R	GGATGCTGCTTAAATCTGATAAAAATA	ChIP
hdac3* P2 *F	AGCTCAAACCGATCTGGTCA	ChIP
hdac3* P2 *R	GAATTTGCTGATCTACTGGG	ChIP
hdac3* P3 *F	TCCGTTTCATCTTTTTAACCTTG	ChIP
hdac3* P3 *R	AGATCCCAGTGCATGAATT	ChIP
hdac3* P4 *F	TGTATGAATTGATTATGTGGTGAAAAT	ChIP
hdac3* P4 *R	ACCCTATAAAGTGGCTGGTGAG	ChIP
hdac3* P5 *F	TGCCAAATTTTTACCCTACCA	ChIP
hdac3* P5 *R	GGGAACTGAGGTTACGATTC	ChIP
hdac3* P6 *F	GACCAATTTCTGGCACCAAC	ChIP
hdac3* P6 *R	TGCAATTTAAACCGAACATGG	ChIP
stat1a* P1 *F	CAACTGGTTTTAGAATAACTTGCAGA	ChIP

stat1a* P1 *R	TCATTGCTTGTTTTATGGAATGTC	ChIP
stat1a* P2 *F	GTCAAAGTCAGGGAGGTGGT	ChIP
stat1a* P2 *R	CTTTGCAACCACACAAAAC	ChIP
stat1a* P3 *F	TGATAAAATAAGCATCTTTTTCTGCT	ChIP
stat1a* P3 *R	AGTGTGGAATTTTGTGCCTG	ChIP
stat1a* P4 *F	AGCACACAAGCTCAAAACACC	ChIP
stat1a* P4 *R	TCGATGAGCAAGTGTGTAGGAA	ChIP
stat1a* P5 *F	CCTGCGGATTCTCACTGTAC	ChIP
stat1a* P5 *R	ACGACGCATTGATCCAACAT	ChIP

F, forward primer; R, reverse primer
